# Are Minimal Radial Distortion Solvers Really Necessary for Relative Pose Estimation?

**DOI:** 10.1007/s11263-025-02657-3

**Published:** 2026-01-09

**Authors:** Viktor Kocur, Charalambos Tzamos, Yaqing Ding, Zuzana Berger Haladova, Torsten Sattler, Zuzana Kukelova

**Affiliations:** 1 Faculty of Mathematics, Physics and Informatics, Comenius University Bratislava, Bratislava, Slovakia; 2 Visual Recognition Group, Faculty of Electrical Engineering, Czech Technical University in Prague, Prague, Czech Republic; 3 Czech Institute of Informatics, Robotics and Cybernetics, Czech Technical University in Prague, Prague, Czech Republic

## Abstract

Estimating the relative pose between two cameras is a fundamental step in many applications such as Structure-from-Motion. The common approach to relative pose estimation is to apply a minimal solver inside a RANSAC loop. Highly efficient solvers exist for pinhole cameras. Yet, (nearly) all cameras exhibit radial distortion. Not modeling radial distortion leads to (significantly) worse results. However, minimal radial distortion solvers are significantly more complex than pinhole solvers, both in terms of run-time and implementation efforts. This paper compares radial distortion solvers with two simple-to-implement approaches that do not use minimal radial distortion solvers: The first approach combines an efficient pinhole solver with sampled radial undistortion parameters, where the sampled parameters are used for undistortion prior to applying the pinhole solver. The second approach uses a state-of-the-art neural network to estimate the distortion parameters rather than sampling them from a set of potential values. Extensive experiments on multiple datasets, and different camera setups, show that complex minimal radial distortion solvers are not necessary in practice. We discuss under which conditions a simple sampling of radial undistortion parameters is preferable over calibrating cameras using a learning-based prior approach. Code and newly created benchmark for relative pose estimation under radial distortion are available at https://github.com/kocurvik/rdnet.

## Introduction

Estimating the relative pose of two cameras, *i.e.*, estimating the relative rotation, translation, and potentially internal calibration parameters of both cameras, is a fundamental problem in computer vision. Relative pose solvers are core components of Structure-from-Motion (SfM) (Wu 2011, Schönberger & Frahm 2016) and localization pipelines (Sattler et al. 2016, Svärm et al. 2016, Sarlin et al. 2019) and play an important role in robotics (Mur-Artal *et al.* 2015, Mur-Artal & Tardós 2017) and autonomous driving (Scaramuzza & Fraundorfer, 2011).

A predominant way to estimate the relative pose of two cameras is based on 2D-2D point correspondences between the two images. Due to noise and the presence of outliers, robust estimation algorithms, such as RANdom SAmple Consensus (RANSAC) (Fischler & Bolles, 1981), or its more modern variants (Raguram et al., 2013; Barath & Matas, 2018), are used for the estimation. Inside RANSAC two different steps are performed: (i) Estimating the camera geometry from a (small or minimal) sample of correspondences and classifying all correspondences into inliers and outliers w.r.t.the obtained camera model. (ii) Local optimization (LO) of the camera model parameters on (a subset of) the inliers to better account for noise in the 2D point positions (Chum et al., 2003; Lebeda et al., 2012).

The main objective of the first step is to obtain a camera geometry estimate and the subset of correspondences consistent with it. Small samples are preferable since the number of RANSAC iterations, and thus the run-time, depends exponentially on the number of correspondences required for model estimation. Solvers that estimate the camera geometry using a minimal number of correspondences and using all available polynomial constraints are known as minimal solvers. The most commonly used minimal solvers for relative pose estimation are the well-known 5-point solver (Nistér 2004) for calibrated cameras and the 7-point solver (Hartley & Zisserman, 2003) for uncalibrated cameras. Both are highly efficient.

Minimal solvers produce estimates that perfectly fit the correspondences in the minimal sample. In practice, the 2D point correspondences are noisy and the noise in the 2D coordinates propagates to the estimates. The goal of the second step, *i.e.*, LO inside RANSAC, is to reduce the impact of measurement noise on pose accuracy (Lebeda et al., 2012). Commonly, a non-minimal solver that fits model parameters to a larger-than-minimal sample is used (Chum et al., 2003), or a robust cost function that optimizes model parameters on all inliers is minimized (Lebeda et al., 2012). The most common non-minimal solver for relative pose estimation is the linear 8-point solver (Hartley & Zisserman, 2003).

All previously mentioned solvers, *i.e.*, the 5-point, 7-point, and 8-point solvers, are widely used in SfM pipelines and other applications. They are based on the pinhole camera model. Yet, virtually all cameras exhibit some amount of radial distortion. Ignoring the distortion, even for standard consumer cameras, can lead to errors in 3D reconstruction (Fitzgibbon, 2001), camera calibration accuracy, *etc.*

There are several ways to deal with radial distortion: (1) Ignore radial distortion estimation during RANSAC and model it only in a post-processing step, *e.g.*, during bundle adjustment in SfM (Snavely et al., 2008; Schonberger & Frahm, 2016). (2) Ignore radial distortion in the first RANSAC step but take it into account in the second step (LO), *e.g.*, by using a non-minimal solver that estimates radial undistortion parameters or by modeling distortion when minimizing a robust cost function. (3) Already estimate the radial distortion in the first RANSAC step (and refine it during LO). Approach (3) is the most principled solution as it enables taking radial distortion into account during inlier counting. Ignoring radial distortion inside the solver typically leads to identifying only the subset of the inliers that is less affected by the distortion.^1^ As a result of only containing points that are only mildly affected by distortion, this inlier set often does not contain enough information to accurately estimate the undistortion parameters. Thus, approaches (1) and (2), which operate on the inliers identified beforehand, are likely to fall into local minima, without recovering correct distortion and camera parameters.

Radial distortion modeling is a mathematically challenging task, and even the simplest one-parameter radial distortion model leads to complex polynomial equations when incorporated into relative pose solvers (Fitzgibbon, 2001; Kukelova & Pajdla, 2007). Thus, algorithms for estimating epipolar geometry for cameras with radial distortion started appearing only after introducing efficient algebraic polynomial solvers into the computer vision community (Fitzgibbon 2001, Barreto & Daniilidis 2005, Jin 2008, Byröd et al. 2009, Kukelova et al. 2010). With improvements in methods for generating efficient polynomial solvers, also minimal radial distortion solvers are improving their efficiency and stability. However, compared to solvers for the pinhole camera model, most of these solvers are still orders of magnitude slower, *e.g.*, the fastest 9-point solver for different distortions runs 210$\mu s$ (Oskarsson, 2021), and the 6-point solver with unknown common radial distortion for calibrated cameras runs 1.18*ms*. This is significantly slower than the 5-point and 7-point pinhole camera solvers that run in less than 6$\mu s$. Moreover, since these solvers estimate more unknown parameters, they need to sample more points inside RANSAC.^2^ They also return more potential solutions to the camera model. Thus, even though radial distortion solvers estimate models that better fit the data, they may require more RANSAC iterations and longer RANSAC run-times.

Radial distortion solvers are not only slower but also more complex to implement. At the same time, many of the existing minimal radial distortion solvers do not have a publicly available implementation. Even though the papers that present novel radial distortion solvers show advantages of these solvers on real data, they usually focus on presenting novel parameterizations and solution strategies and their numerical stability on synthetic data. Real experiments are mostly limited to small datasets (*e.g.*, a single scene), simpler variants of RANSAC, and qualitative instead of quantitative results. The above-mentioned facts are most likely the reasons why (minimal) radial distortion solvers are not often used in practice. Instead, it is common to use either approach (1) or (2) (Schonberger & Frahm, 2016; Snavely et al., 2008). Naturally, this leads to the question whether (minimal) radial distortion solvers are actually necessary in practical applications.

The goal of this paper is to answer this question. To this end, we introduce two new approaches to relative pose estimation under radial distortion: (1) We introduce a new sampling-based strategy that combines an efficient solver for uncalibrated relative pose problem (e.g. the 7-point $\textbf{F}$ solver (Hartley & Zisserman, 2003), or the 6-point $\textbf{E}f$ solver (Larsson et al., 2017)) with a sampled undistortion parameter: In each RANSAC iteration, we run the solver potentially several times (1-3x) with different (but fixed) undistortion parameters. (2) Rather than sampling from a fixed set of undistortion parameters, the second approach uses a learning-based prior for the parameters: A neural network (Veicht et al., 2024) predicts the radial undistortion (and potentially other camera) parameters, which are then used to obtain initial pose estimates inside RANSAC. The paper makes the following contributions:

1. We extensively evaluate different approaches for uncalibrated relative pose estimation under radial distortion on multiple datasets, under different scenarios. We are not aware of any such practical evaluation of radial distortion solvers in the literature.
2. We show that both the sampling-based and learning-based prior strategies, which are both easy to implement, perform similar or better than most accurate radial distortion solvers. We thus show that dedicated minimal radial distortion solvers for the relative pose problem are not necessary in practice.
3. We show that for the case of two cameras with unknown and shared intrinsics, the sampling-based strategy combined with the 6-point $\textbf{E}f$ solver (Larsson et al., 2017) provides similar accuracy to the learning-based prior approach while its total runtime is lower and it does not require a GPU to run efficiently.
4. For the case of two cameras with different intrinsics, we show that the simple sampling-based strategy performs better than other methods when the time budget for computation is limited (<100 ms) or the computation has to be performed on low-cost hardware.
5. We show that the sampling-based strategy is more robust and works for all types of data compared to learning-based priors that can be less precise for some types of data (*e.g.*images outside of the training distribution).
6. We create a new benchmark, consisting of two scenes, containing images taken with different cameras with multiple different distortions.
7. Code and dataset are available at https://github.com/kocurvik/rdnet.

This paper is an extension of our previous work (Charalambos et al., 2024). Compared to (Charalambos et al., 2024), this work strengthen the main message of the original work, namely that minimal radial distortion solvers are not necessary, by (1) including a new approach to uncalibrated relative pose estimation under radial distortion that does not rely on a minimal radial distortion solver (the learning-based prior strategy introduced in Sec. 3.5); (2) performing experiments on two additional datasets (PragueParks (Jin et al., 2020) and EuRoc-MaV (Burri et al., 2016)) from the literature, significantly increasing the number of image pairs that are used for evaluation by 13.7k pairs; (3) evaluating additional minimal solvers for relative pose estimation together with our two strategies (sampling-based and learning-based prior-based approaches); and (4) providing insights into which of our strategies is preferable under which conditions.

## Related Work

The literature studies three groups of radial distortion relative pose problems: Two cameras with equal and unknown radial distortion; two cameras where only one has unknown distortion; two cameras with different and unknown distortion.

### Equal and unknown radial distortion

Fitzgibbon (Fitzgibbon, 2001) introduced an one-parameter division model for modeling undistortion and an algorithm for estimating the fundamental matrix with equal and unknown radial distortion using this model. This algorithm does not use the singularity constraint on the fundamental matrix, necessitating 9 point correspondences instead of the minimal 8. This approach transforms the problem into a standard quadratic eigenvalue problem with up to 10 solutions. The first minimal solution for epipolar geometry estimation with the one-parameter division model using 8 point correspondences was proposed by Kukelova and Pajdla (2007), using the Gröbner basis method (Cox et al., 2005) to solve a system of polynomial equations. This solver has been improved by using an automatic generator of Gröbner basis solvers (Kukelova et al., 2008), performing Gauss-Jordan (G-J) elimination of a 32$\times$48 matrix and eigenvalue decomposition of a 16$\times$16 matrix, which has up to 16 solutions. Jiang et al. (2015), used 7 point correspondences to solve the problem of essential matrix estimation for two cameras with equal and unknown focal length and radial distortion. This problem results in a complex system of polynomial equations and a large solver that performs the LU decomposition of an 886$\times$1011 matrix and computes the eigenvalues of a 68$\times$68 matrix. Thus, this solver is too time-consuming for practical applications. A similar but more efficient solver was proposed by Oskarsson (2021), however the solver is highly unstable making it impractical.

### One unknown radial distortion

Kuang et al. (2014) studied three minimal cases for relative pose estimation with a single unknown radial distortion based on the Gröbner basis method: 8-point fundamental matrix and radial distortion; 7-point essential matrix, focal length and radial distortion; 6-point essential matrix and radial distortion. However, these solvers assume one of the two cameras has known or no radial distortion. In many scenarios, this assumption does not hold.

### Different and unknown radial distortions

All of the above mentioned algorithms estimate only one radial undistortion parameter for one or both cameras. In practice, *e.g.*, using images downloaded from the Internet, two cameras can have different and unknown radial distortions. The problem of fundamental matrix estimation with different and unknown radial distortions, $\textbf{F} \lambda _1 \lambda _2$, was first studied by Barreto and Daniilidis (2005), proposing a non-minimal linear algorithm using 15 point correspondences (F15). The minimal 9-point case (F9) for this problem was studied in Kukelova and Pajdla (2007),Byröd et al. (2008),Kukelova et al. (2008),Kukelova et al. (2010). The solver from Byröd et al. (2008) (F9) performs LU decomposition of a 393$\times$389 matrix, SVD decomposition of a 69$\times$69 matrix, and eigenvalue computation of a 24$\times$24 matrix. In Kukelova et al. (2008), a faster version based on a Gröbner basis ($\mathrm F9_\textrm{A}$) was proposed. It performs G-J elimination of a 179$\times$203 matrix and eigenvalue decomposition of a 24$\times$24 matrix. However, this solver is slightly less stable than F9, and still too slow for real-time applications. Kukelova et al. (2010) suggested an efficient, non-minimal solver using 12 point correspondences (F12) that generates up to four real solutions. However, this algorithm is more sensitive to noise than the minimal $\mathrm F9_\textrm{A}$. Balancing efficiency and noise sensitivity, Kukelova et al. (2015) proposed a 10-point solver that is much faster than the minimal 9-point solver and more robust to image noise than the 12-point solver.

Recently, Oskarsson (2021) presented a unified formulation for relative pose problems involving radial distortion and proposed more efficient minimal solvers for all different configurations. While some of the proposed solvers are already quite efficient, *e.g.*, the 8-point solver for uncalibrated cameras with common radial distortion, others, like the 9-point solver for different distortions, are still too slow and/or numerically unstable to be useful in practice.

### Parameter sampling

Instead of jointly estimating the absolute camera pose and the focal length of an uncalibrated camera, Sattler et al. (2014) proposed a RANSAC variant that combines parameter sampling and parameter estimation. In each RANSAC iteration, they first randomly sample a focal length value and then estimate the pose of the now-calibrated camera. The probability distribution over the focal length values is then updated based on the number of inliers of the estimated pose. We propose a simpler sampling-based strategy for relative pose estimation that uses a small fixed set of undistortion parameters. In contrast to Sattler et al. (2014), our approach can easily be applied to 2D sampling problems, *e.g.*, two different and unknown undistortion parameters.

## Radial Distortion Estimation

**Fig. 1:**
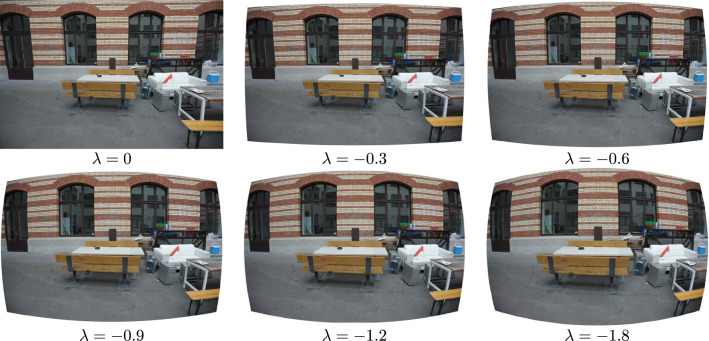
Example image of the Courtyard scene from the ETH3D dataset, w.r.t. synthetically added distortions. We use undistortion parameters $\{0, -0.3, -0.6, -0.9, -1.2, -1.8\}$ and compute the distorted images using the inverse of the one-parameter division undistortion model. For the parameters, we consider the one-parameter division model and $[-0.5, 0.5]$ coordinate normalization

### Background

A pair of corresponding distorted image points $\textbf{x}_i \leftrightarrow \textbf{x}_i^\prime$, detected in two uncalibrated images, is related by the epipolar constraint

1 $$\begin{aligned} u(\textbf{x}_i, \mathbf {\Lambda })^\top \textbf{F} u(\textbf{x}_i^\prime , \mathbf {\Lambda }^\prime ) = 0 \hspace{5.0pt}, \end{aligned}$$

where $\textbf{x}_i, \textbf{x}_i^\prime \in \mathbb {P}^2$, $\textbf{F}$ is the fundamental matrix encoding the relative pose and the internal calibrations of the two cameras, and $u:\mathbb {P}^2 \times \mathbb {R}^{n} \rightarrow \mathbb {P}^2$ denotes an undistortion function, which is a function of the distorted image point $\textbf{x}_i$ and *n* undistortion parameters $\mathbf {\Lambda } \in \mathbb {R}^{n}$.

In this paper, we model the undistortion function using the one-parameter division model (Fitzgibbon, 2001). In this model, given an observed radially distorted point with homogeneous coordinates $\textbf{x} = [x_d, y_d, 1]^\top$, and the undistortion parameter $\lambda \in \mathbb {R}$, the undistorted image point is given as

2 $$\begin{aligned} u(\textbf{x}, \lambda ) = [x_d, y_d, 1 + \lambda (x_d^2 + y_d^2)]^\top \hspace{5.0pt}, \end{aligned}$$

assuming that the distortion center is in the image center. This model is very commonly used in practice due to its simplicity, efficiency, and robustness, since it can adequately capture even large distortions of wide-angle lenses. It is incorporated in almost all minimal and non-minimal radial distortion solvers. The values of $\lambda$ in (2) are expressed relative to the image size. To keep the values consistent across images, we normalize the image coordinates so that the longer side covers the range $\left[ -0.5, 0.5 \right]$. Under such a normalization, the $\lambda$ values fall within the range $\left( -2.0, 0.0 \right]$. The effect of different values of $\lambda$ is shown in Fig. 1.

### Radial Distortion Solvers

The goal of this paper is not to introduce novel minimal or non-minimal radial distortion solvers, but to study the performance of the existing solvers under different conditions. We study the two most practical scenarios of two uncalibrated cameras with unknown (i) equal and (ii) different radial distortions. We denote these problems as (i) the $\textbf{F} \lambda$ and (ii) the $\textbf{F} \lambda _1\lambda _2$ problems.^3^ Next, we briefly describe the radial distortion solvers for these two problems studied in this paper, as well as some improvements to these solvers.

$\textbf{F} \lambda$ : Assuming equal unknown distortion modeled using the one-parameter division model (2), the relative pose problem for uncalibrated cameras has 8 degrees of freedom (DoF). For this problem, we consider the following solvers:

- 8pt $\textbf{F} \lambda$ : Among all minimal 8pt solvers (Kukelova & Pajdla, 2007; Kukelova et al., 2008; Larsson et al., 2017; Oskarsson, 2021), the solver from Oskarsson (2021) is the most efficient. It formulates the elements of $\textbf{F}$ as functions of the undistortion parameter $\lambda$, and obtains an univariate polynomial in $\lambda$ of degree 16, which can be solved using the Sturm sequences, with up to 16 solutions.
- 9pt $\textbf{F} \lambda$ : By ignoring the $\det (\textbf{F}) = 0$ constraint, the $\textbf{F} \lambda$ problem can be solved using nine point correspondences. Fitzgibbon (2001) solves the nine equations (1) by converting them into a polynomial eigenvalue problem. While (Fitzgibbon, 2001) was able to remove several spurious solutions by transforming the original eigenvalue problem of size $18 \times 18$ into a problem of size $10 \times 10$, Fitzgibbon (2001) also observed that 4 of the 10 solutions of this system are imaginary. In this paper, we propose a modification of the solver proposed in Fitzgibbon (2001), in which we directly remove 4 imaginary solutions, resulting in a more efficient solver that needs to find the eigenvalues of a smaller $6\times 6$ matrix. To remove these 4 imaginary solutions, we use the structure of matrices that appear in the polynomial eigenvalue formulation of this problem and the method proposed in Kukelova et al. (2012). For more details, see Sec. 3.3.1.

$\textbf{F} \lambda _1\lambda _2$ : For the case of different unknown radial distortions, we have 9 DoF. For this problem, we consider the following solvers:

- 9pt $\textbf{F} \lambda _1\lambda _2$ : Equations for cameras with different unknown distortions are more complex than for the equal distortion case. In this case the system of equations has 24 solutions and the fastest Gröbner basis solver from Larsson et al. (2017), which returns 24 solutions, performs elimination of a large matrix of size $84 \times 117$ followed by the eigendecomposition of a $24 \times 24$ matrix. The recently published parameterization of this problem in Oskarsson (2021) performs elimination of a smaller matrix of size $51 \times 99$ followed by the eigendecomposition of a $48 \times 48$ matrix. The solver returns up to 48 solutions. However, it is still faster than the solver from Larsson et al. (2017). Thus, in our experiments, we use the solver from Oskarsson (2021).
- 10pt $\textbf{F} \lambda _1\lambda _2$ : In Kukelova et al. (2015) it was shown that in many scenarios inside RANSAC it is preferable to sample 10 instead of 9 points and run the more efficient 10pt solver. In Kukelova et al. (2015), the authors proposed several variants of the 10pt solver. In this paper, we use the variant based on a Gröbner basis, made available by the authors. The 10pt solvers cannot be easily modified to work with more than 10 points, and thus we use this solver only in the first step of RANSAC, *i.e.*, instead of the minimal solver, and not in the LO step.

### Modified Solver for Non-minimal Fitting

In this section, we describe the proposed modification to the polynomial eigenvalue 9pt $\textbf{F} \lambda$ solver, in which we remove spurious solutions. We also discuss how to extend these solvers to work with more points.

#### $\textbf{F} \lambda$ solver for equal and unknown distortion

Based on (Fitzgibbon, 2001), the epipolar constraint with equal and unknown radial distortion can be written as

3 $$\begin{aligned} \begin{array}{ccccccccccccccccc} & & [ & x'_d x_d & x'_d y_d & x'_d & y'_d x_d & y'_d y_d & y'_d & x_d & y_d & 1 & ]& \cdot & \textbf{f} & \\ \quad +& \lambda & [ & 0 & 0 & x_d r^{2} & 0 & 0 & y'_d r^{2} & x_d r^{\prime 2} & y_d r^{\prime 2} & r^{2}+r^{\prime 2} & ]& \cdot & \textbf{f} & \\ \quad + & \lambda ^2 & [ & 0 & 0 & 0 & 0 & 0 & 0 & 0 & 0 & r^{2} r^{\prime 2} & ]& \cdot & \textbf{f} & = & 0 \hspace{5.0pt}, \end{array} \end{aligned}$$

where $\textbf{f}$ is a $9\times 1$ vector that contains the entries of the fundamental matrix $\textbf{F}$ in row-wise order and $r, r^\prime$ denote the Euclidean distances of the distorted points $\mathbf {x_i}, \mathbf {x_i^\prime }$, respectively, to the center of distortion. It is common to assume that the center of distortion is in the center of the image, *i.e.*, $r = \sqrt{x_d^2 + y_d^2}$.

For n point correspondences, (3) can be written in a matrix form

4 $$\begin{aligned} (\textbf{A}_0 + \lambda \textbf{A}_1 + \lambda ^2 \textbf{A}_2)\textbf{f} = \textbf{0} \hspace{5.0pt}, \end{aligned}$$

where $\textbf{A}_0,\textbf{A}_1$ and $\textbf{A}_2$ are $n \times 9$ coefficient matrices. For 9 point correspondences in the 9pt $\textbf{F} \lambda$ solver, equation (4) defines a polynomial eigenvalue problem that can be solved by computing the eigenvalues of a $18 \times 18$ matrix. In Fitzgibbon (2001), it was shown how the number of solutions of (4) can be reduced from 18 to 10 by transforming the problem to an eigenvalue problem of size $10 \times 10$. However, in Fitzgibbon (2001) it was also noted that 4 of these 10 solutions have been found to be imaginary. In our case, we show that the 4 imaginary solutions can be directly removed and we only need to find the eigenvalues of a $6\times 6$ matrix. Since matrix $\textbf{A}_2$ is singular while $\textbf{A}_0$ is full-rank, we first let $\sigma = 1/\lambda$. Then (4) can be written as

5 $$\begin{aligned} (\textbf{A}_2 + \sigma \textbf{A}_1 + \sigma ^2 \textbf{A}_0)\textbf{f} = \textbf{0} \hspace{5.0pt}. \end{aligned}$$

Solving for $\sigma$ is equivalent to finding the eigenvalues of the following $18 \times 18$ matrix

6 $$\begin{aligned} \textbf{B} = \begin{bmatrix} \textbf{0} & {\textbf{I}}\\ -{\textbf{A}}_0^{-1}{\textbf{A}}_2 & -{\textbf{A}}_0^{-1}{\textbf{A}}_1 \end{bmatrix} \hspace{5.0pt}. \end{aligned}$$

There are 8 zero columns in $\textbf{A}_2$, and 4 zero columns in $\textbf{A}_1$. To solve this problem efficiently, we use the technique from Kukelova et al. (2012): the zero columns in $-{\textbf{A}}_0^{-1}{\textbf{A}}_2$ and $-{\textbf{A}}_0^{-1}{\textbf{A}}_1$ can be removed together with the corresponding rows. In this case, the size of the matrix $\textbf{B}$ is reduced to $6\times 6$, and we only need to find the eigenvalues of a $6\times 6$ matrix. Note that in the solver, we directly construct the reduced $6 \times 6$ matrix and avoid computations on the matrix (6)

For the non-minimal case, *i.e.*, the case where the number of point correspondences is larger than 9, $-{\textbf{A}}_0^{-1}{\textbf{A}}_2$ and $-{\textbf{A}}_0^{-1}{\textbf{A}}_1$ are solved using linear least squares (which can be efficiently solved using the ColPivHouseholderQR function in the Eigen library (Guennebaud et al., 2010)).

### Sampling Distortions

While the non-mininmal 9pt $\textbf{F} \lambda$, and 10pt $\textbf{F} \lambda _1\lambda _2$ solvers are reasonably efficient, the minimal 8pt $\textbf{F} \lambda$ and especially the 9pt $\textbf{F} \lambda _1\lambda _2$ solver are significantly slower than the minimal uncalibrated 7pt pinhole camera solver (Hartley & Zisserman, 2003) or even a slightly more complex 6pt $\textbf{E}f$ solver (Larsson et al., 2017) for pinhole cameras with common unknown focal length. Moreover, the minimal radial distortion solvers return more solutions, *i.e.*, 16, 24, or even 48 compared to the 3 solutions of the 7pt solver and 15 solutions of the 6pt $\textbf{E}f$ solver (Larsson et al., 2017). More solutions lead to reduced efficiency, since within a RANSAC framework each solution has to be evaluated. This, together with the fact that the radial distortion solvers sample more points and solve significantly more complex equations, motivates a common strategy in which in the first step of RANSAC, a solver for pinhole camera without radial distortion, usually the standard 7pt solver, is applied, and the radial distortion is modeled only in the LO step of RANSAC.

However, as mentioned in Sec. 1, for images with larger distortion, the standard perspective camera model without distortion may not properly model the data and may thus not return a large-enough subset of the true inliers and/or an accurate-enough initial pose estimate. Yet, small changes in the undistortion parameter $\lambda$ in (2), in general, do not result in large changes in the projection of points into the image. For an undistortion parameter $\lambda$ that is reasonably close to the true parameter $\lambda _\text {true}$, we can thus expect that applying the 7pt solver on 2D point positions that were undistorted using $\lambda$ can result in sufficiently-large inlier sets and sufficiently-accurate initial poses that will lead to good estimates in the LO step.

The discussions above motivate a simple sampling-based strategy that we propose in this paper: In each iteration of RANSAC, it runs the standard 7-point $\textbf{F}$ solver (Hartley & Zisserman, 2003) or the 6-point $\textbf{E}f$ solver (Larsson et al., 2017) on image points undistorted with a fixed radial undistortion parameter sampled from an interval of feasible undistortion parameters. In this approach, we use the facts that the 7pt $\textbf{F}$ and the 6pt $\textbf{E}f$ solvers are significantly faster than the minimal radial distortion solvers, and return fewer solutions that need to be tested inside RANSAC. Thus, even running the 7pt $\textbf{F}$ or the 6pt $\textbf{E}f$ solvers several times with different fixed undistortion parameters in each RANSAC iteration may lead to a higher efficiency compared to running the 8pt $\textbf{F} \lambda$ or 9pt $\textbf{F} \lambda _1\lambda _2$ radial distortion solvers.

The best choices for the number *k* of runs of the 7pt $\textbf{F}$ or the 6pt $\textbf{E}f$ solver in each iteration, and the values $\textbf{U}_i = \{\hat{\lambda }_i^1, \hat{\lambda }_i^2,..., \hat{\lambda }_i^k\}$, which are used to undistort points in the two cameras $i=1,2$, can differ depending on the application and input data. In our experiments, we test three variants of the sampling solver: (1) $\textbf{U}_1 = \textbf{U}_2 = \{ 0 \}$, which represents the above mentioned standard baseline that assumes no distortion in the first step of RANSAC; (2) $\textbf{U}_i = \{ \hat{\lambda }_i \}$, $i=1,2$ where we run the 7pt $\textbf{F}$ or the 6pt $\textbf{E}f$ solver only once for one fixed value of $\hat{\lambda }_i \ne 0$. This can represent a scenario where we have prior knowledge that our images have visible distortion. In our experiments, we test a version with $\hat{\lambda }_i$ that represents medium distortion and can potentially, after LO, provide good results even for cameras with small or large distortion; (3) $\textbf{U}_i = \{\hat{\lambda }_i^1, \hat{\lambda }_i^2, \hat{\lambda }_i^3\}$, where we undistort points in each camera with three different fixed parameters that represent, *e.g.*, small, medium, and large distortion. This setup is, *e.g.*, useful in scenarios where we have images from the “wild" (*e.g.*, the Internet) that can have a wide variety of different distortions. Note that in this case, if we assume cameras with different distortions, we test only the uncalibrated 7pt $\textbf{F}$ solver, and we run this solver nine times. Still, this is more efficient than using the dedicated distortion $\textbf{F} \lambda _1\lambda _2$ or $\textbf{F} \lambda$ solvers.

### Learning-based Priors for Radial Distortion Estimation

The solvers discussed in Sections 3.2 and 3.3 estimate the radial undistortion parameter(s) from point correspondences. In contrast, our sampling-based approach uses manually selected priors for the undistortion parameters, which can then be refined during LO. Rather than manually selecting these priors, radial distortion parameters, as well as other intrinsic parameters such as the focal length, can also be inferred from a single or multiple images via explicit geometric cues (Lochman et al., 2021), or by using learning-based approaches (Jin et al., 2023; Veicht et al., 2024). To answer the question whether (minimal) radial distortion solvers are necessary in practical applications, we thus also evaluate a strategy that uses radial distortion and intrinsic priors obtained via learning rather than using manually selected radial distortion priors or estimating radial distortion from point correspondences.

We use GeoCalib (Veicht et al., 2024), a recent, state-of-the-art end-to-end deep learning approach that predicts camera intrinsics (focal length and a radial undistortion parameter) and the gravity direction from a single image or multiple images. GeoCalib first employs a convolutional neural network to infer visual cues in the form of a Perspective Field (Jin et al., 2023), storing per-pixel up-vector and latitude estimates and their uncertainties. The camera parameters that model the observations stored in this Perspective Field are then found using differentiable Levenberg-Marquart (LM) optimization. If multiple images produced by a single camera are available, the shared intrinsics can be estimated jointly from the Perspective Fields of all images, resulting in better accuracy.

GeoCalib simultaneously predicts the camera’s focal length, a radial undistortion parameter for one-parameter division model, and the gravity direction. These camera parameter predictions can be used within a RANSAC pipeline as prior information, similarly to how the sampled undistortion parameters are utilized. We use these priors in three ways: (1) we only use the predicted radial undistortion parameter, instead of a sampled parameter, and run the standard 7pt $\textbf{F}$ or 6pt $\textbf{E}f$ solvers on image points undistorted by the predicted undistortion parameter. (2) we use both the predicted radial undistortion parameter and the focal length as priors. We use the focal length to calibrate the image points, and the undistorion parameter to undistort them. We then run the 5pt solver for calibrated pinhole cameras. (3) we use all predicted parameters to run the 3pt solver (Ding et al., 2023, 2020) that estimates the relative pose between two cameras with known gravity directions.

Compared to our sampling strategy, using learning-based priors has the potential to speed up the estimation process: (1) The inferred undistortion parameter can be closer to the ground truth parameter than the sampled one(s). (2) We only need to test a single parameter compared to several undistortion parameters that usually have to be evaluated by sampling-based strategy. (3) The inferred focal lengths and gravity directions simplify the relative pose problems that need to be solved. However, a modern GPU is needed for GeoCalib, whereas the sampling strategy only requires CPU-based computations. Thus, the approach that uses learning-based priors might not always be applicable, *e.g.*, it requires too much resources for robotics and on-device augmented reality applications, where energy consumption and battery capacity are limiting factors. Furthermore, the predictions by GeoCalib might not be accurate, especially if the input image(s) is very different from GeoCalib’s training data. In such cases, our sampling-based strategy will still perform well.

## Experiments

### Datasets

We evaluate the different approaches for radial distortion relative pose estimation on four datasets: ETH3D (Schops et al., 2017), PragueParks  (Jin et al., 2020), EuRoC-MAV  (Burri et al., 2016), and our new benchmark, each covering different scenarios.

The ETH3D dataset was designed to evaluate (multi-view) stereo algorithms (Schops et al., 2017). It covers indoor and outdoor scenes captured with a DSLR camera. Ground truth poses were obtained by aligning the images to high-precision laser scans. ETH3D provides undistorted images together with their intrinsic calibration. We use 2,037 image pairs from 12 ETH3D scenes.

The PragueParks dataset (Jin et al., 2020) contains images extracted from iPhone 11 video sequences, in which both standard and wide-angle lenses are used. The authors provide ground truth poses obtained using RealityCapture SfM software (Capturing Reality, 2024). The dataset features vegetation-rich scenes such as trees, ponds, sculptures, with different levels of zoom, and no people. We use 453 pairs of undistorted images from 3 PragueParks scenes. We use undistorted images from ETH3D and PragueParks datasets in experiments, where we synthetically distort them to simulate different scenarios, *e.g.*scenario with images with different distortions downloaded from the Internet.

The EuRoC-MAV dataset (Burri et al., 2016) is a widely used benchmark for visual-inertial odometry and SLAM, captured using a Micro Aerial Vehicle equipped with synchronized stereo cameras and an IMU. It features sequences recorded in indoor environments such as machine halls and office spaces, with varying levels of motion dynamics and lighting conditions. The dataset provides accurate ground truth from a motion capture system, making it ideal for evaluating pose estimation algorithms. We use 13,315 image pairs from 6 EuRoC-MAV scenes. The provided ground truth parameters are based on a radial-tangential distortion model rather than the division model, therefore, distortion error is not reported for this dataset.

### New Benchmark

Existing datasets (Burri et al., 2016; Schops et al., 2017) containing radially distorted images mostly involve only one or two different camera lenses with little variation in the undistortion parameters. Testing the sampling-based strategy on such images could be biased, as it would have been as good as the distance of the used sampled value from the one/two ground truth values. We thus created a new benchmark with a higher variation in the undistortion parameters, consisting of two scenes: ROTUNDA and CATHERAL. For both scenes, we build upon previously recorded images (Kukelova et al., 2015; Sattler et al., 2019), taken by GoPro cameras and kindly provided by the authors. For both scenes, we recorded additional images with different cameras and, in addition, we downloaded some images from Flickr that depict CATHEDRAL scene. To obtain ground truth poses and camera intrinsics including radial distortion for both the original and the newly added images, we used the RealityCapture software (Capturing Reality, 2024). We configured RealityCapture to estimate the undistortion parameters for the images using the one-parameter division model that we use in all of our experiments, *i.e.*, RealityCapture directly provides ground truth estimates for the undistortion parameters. We enforced that images taken by the same camera (with the same field of view) share the same intrinsic camera parameters. In the following, we briefly describe both scenes.

**Fig. 2:**
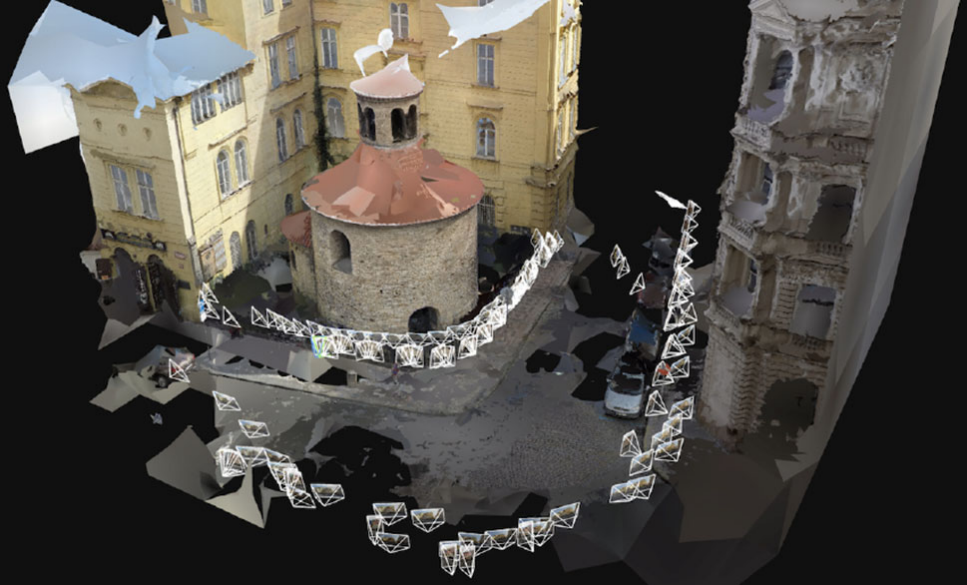
Visualization of the ROTUNDA scene. We show a textured mesh of the scene to provide a clearer visualization. We also show the poses of the 157 images of the dataset

**Fig. 3:**
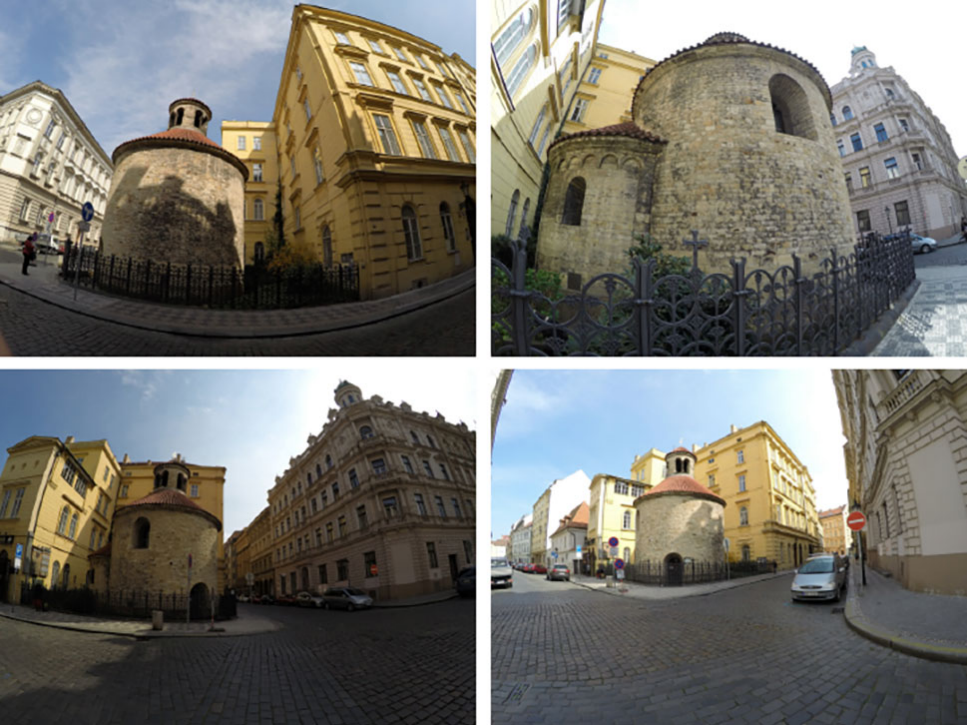
Example images from the ROTUNDA scene

The ROTUNDA scene contains 157 outdoor images of a historical building captured by two mobile phone cameras (95 new images in total) and one GoPro camera (62 images, provided by the authors of Kukelova et al. (2015)). The GoPro images were captured using two different settings that affect the field-of-view and image distortion. Overall, images were taken with four different $\lambda$ values: $\left\{ -1.50, -0.81, 0.02, 0.09\right\}$ (ground truth values provided by RealityCapture after normalization). Fig. 2 visualizes the ROTUNDA scene by showing a textured mesh of the scene together with the camera poses of the recorded images. Figure 3 shows example images from the ROTUNDA scene.

**Fig. 4:**
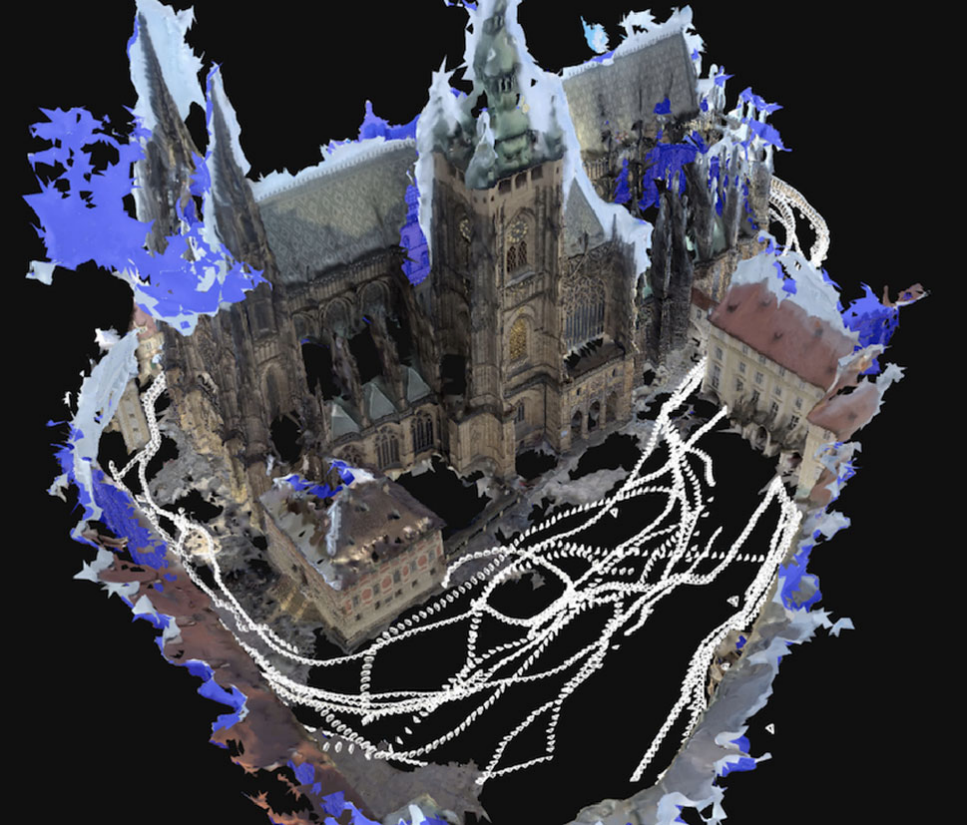
Visualization of the CATHEDRAL scene. We show a colored mesh of the scene to provide a clearer visualization. We also show the poses of the 2,734 images of the dataset

**Fig. 5:**
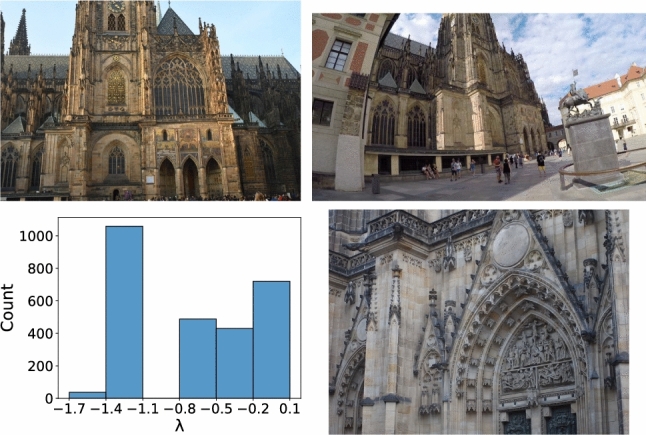
Example images from the CATHEDRAL scene. Distribution of $\lambda$ radial distortion parameters for the CATHEDRAL scene (a). The parameters were obtained by normalizing the ground truth parameters estimated by RealityCapture

The CATHEDRAL scene contains 2,734 outdoor images of a historical cathedral, captured by two mobile phone cameras (708 new images in total), one GoPro camera (655 images, provided by the authors of Sattler et al. (2019)), and an Insta360 Ace Pro camera (1,358 new images). Most of the images were extracted from videos captured while walking around the building. In addition, we are using 13 images from Flickr. The dataset contains images from cameras with 42 different $\lambda$ parameters. Their distribution is shown in Fig. 5 along with example images for the CATHEDRAL scene. Fig. 4 visualizes the CATHEDRAL scene by showing a colored mesh of the scene together with the camera poses of the recorded images.

For our experiments, we use 3,424 image pairs with two different cameras (denoted as $\lambda _1 \ne \lambda _2$) and 1,795 image pairs captured by the same camera and thus with the shared intrinsics (denoted as $\lambda _1 = \lambda _2$) for ROTUNDA scene and 10,000 sampled image pairs for both $\lambda _1 \ne \lambda _2$ and $\lambda _1 = \lambda _2$ for CATHERAL scene.

### Evaluation measures

Following (Jin et al., 2020), given the ground truth and the estimated relative pose, we measure the rotation error and the translation error. The rotation error is defined as the angle of the rotation matrix aligning the estimated with the ground truth rotation matrix. The translation error is defined as the angle between the estimated and the ground truth translation vector. Finally, the pose error is defined as the maximum of the rotation error and the translation error. We also measure the distortion error $\epsilon (\lambda )$ as the absolute distance between ground truth and estimated undistortion parameters and the focal length error as $\xi (f) = \frac{|f_{gt} - f|}{f_{gt}}$, where *f* is the estimated focal length and $f_{gt}$ is the ground truth. For the problem with two different cameras, we measure the distortion error as $0.5 \cdot (\epsilon (\lambda _1)+\epsilon (\lambda _2))$ and the focal length error as $0.5 \cdot (\xi (f_1) + \xi (f_2))$. We report the average (AVG) and median (MED) pose errors, as well as the area under the recall curve (AUC) at a $10^\circ$ threshold on the pose error.

### Experimental setup

We obtained the correspondences between the images by matching SuperPoint (DeTone et al., 2018) features extracted on the images without resizing. We only kept at most 2048 features and matched them with LightGlue (LG) (Lindenberger et al., 2023). We only considered images with sufficient overlap quantified by the co-visibility constraint proposed in Jin et al. (2020). We retained only those image pairs that yielded a minimum of 20 matches. For EuRoC-MAV we kept at most 4096 features to ensure a sufficient number of matches as this indoor dataset is relatively textureless and more challenging.

We evaluate the 8pt $\textbf{F} \lambda$(Oskarsson, 2021), 9pt $\textbf{F} \lambda _1\lambda _2$ (Oskarsson, 2021), and the non-minmal 9pt $\textbf{F} \lambda$ (*cf.*Sec. 3.3.1), and 10pt $\textbf{F} \lambda _1\lambda _2$ (Kukelova et al., 2015) solvers (*cf.*Sec. 3) in RANSAC, and the sampling strategies that combine the 6pt $\textbf{E}f$ (Larsson et al., 2017), and the 7pt $\textbf{F}$ (Hartley & Zisserman, 2003) solver with a set of pre-defined undistortion parameters (*cf.*Sec. 3.4). We denote the latter by appending the list of parameters, *e.g.*, $\{0, -0.6, -1.2\}$, to the solver configuration. Additionally, we evaluate the learning-based prior strategy which combines the 7pt $\textbf{F}$ (Hartley & Zisserman, 2003), 6pt $\textbf{E}f$ (Larsson et al., 2017), 5pt $\textbf{E}$ (Nistér 2004), and 3pt $\textbf{E}$ (Ding et al., 2023, 2020) solvers with the camera parameters predicted using GeoCalib (*cf.*Sec. 3.5). The GeoCalib predictions are obtained by running the network with its default inference settings, followed by 30 iterations (which is the default number of iterations) of LM optimization to refine the estimated camera intrinsics and gravity direction. For the case when the two cameras share intrinsics ($\lambda _1\,=\,\lambda _2$), we utilize GeoCalib’s multi-image optimization setting, which jointly optimizes the shared focal length and radial undistortion parameter of a pair of images while independently refining the gravity direction of each image. In contrast, for the case of two different cameras ($\lambda _1\,\not =\,\lambda _2$), each image is processed independently, and camera parameters are predicted using the default single-image inference pipeline of GeoCalib. GeoCalib inference with the multi-image optimization setting for pairs of images and 30 iterations runs on average $\sim 380 ms$, while for a single-image, inference runs on average $\sim 185ms$ on an NVIDIA A100 (a high-end server grade GPU). We also add an ablation study that analyzes the impact of the number of iterations of LM optimization on run-time and pose estimation accuracy.

We integrate the solvers and strategies into Larsson (2020). The LO step in PoseLib relies on Levenberg-Marquardt (LM) optimization of the truncated Tangent Sampson Error (Terekhov & Larsson, 2023), starting from the estimate provided by the minimal solver, sampling strategy, or the learning-based prior. The pose and intrinsics returned by solvers are further polished by LM optimization over all inliers. For the learning-based prior strategy we evaluate two different LO settings. We either optimized all camera parameters, or leave the parameters estimated by GeoCalib fixed. For each method we denote which parameters were refined in LO. For solvers which produce the fundamental matrix we decompose it using the Bougnoux formula for two different cameras (Bougnoux, 1998) and the Sturm’s formula for cameras with shared intrinsics (Sturm, 2001) into the pose and the focal lengths. We use the closed-form formulas due to their speed since fundamental matrices need to be decomposed for each provided solution. For both decomposition and local optimization we assume that the principal point is fixed in the image center.

To determine which points are inliers we use the Tangent Sampson Error (Terekhov & Larsson, 2023) with a fixed 3px threshold. Using the Tangent Sampson Error is important since the standard Sampson Error in undistorted images leads to a radial bias in the optimization (Terekhov & Larsson, 2023).

We use normalized image coordinates from the range $[-0.5, 0.5]^2$, obtained by subtracting the image center and dividing by the length of the longer image side. For this normalization, the undistortion parameter should be greater than $-2$, as otherwise the distortion would mirror the image. In RANSAC, we thus discard models with radial distortion outside of the meaningful range $\left[ -2.0, 0.5\right]$. Extreme fisheye lenses cannot be modeled by the division model alone. However, larger distortions could be handled using the division model (and minimal solvers based on this model) to obtain an initial estimate in RANSAC. This estimate can then refined using a more complex fisheye model during local optimization inside RANSAC, *e.g.*, using non-linear optimization or a non-minimal solver for a more complex distortion model. We see an advantage for the sampling-based strategy when a more complex distortion model needs to be used during the initialization step: There is no need for minimal solvers for such models (which, to the best of our knowledge, do not exist for more complex distortion models). Instead, sampling can be directly used to undistort the measurements using a complex distortion model, followed by applying the 7pt $\textbf{F}$ solver. Note that more complex distortion models will likely require more samples, which in lead to longer run-times as more camera pose hypotheses would be created and evaluated. Investigating the effect of different distortion models and local optimization strategies on images recorded with extremely distorted lenses is beyond the scope of this paper and is left for future work.

### Prior knowledge about cameras

For the sampling-based strategy, we can adjust the number *k* of samples and the sampled values $\textbf{U}_i = \{\hat{\lambda }_i^1, \hat{\lambda }_i^2,..., \hat{\lambda }_i^k\}$ based on prior knowledge about the cameras. We study three different scenarios.

#### Scenario A - *Wild*

In the first scenario, we assume no knowledge about the cameras. The cameras can have distortions ranging from small to very high. This scenario represents, *e.g.*, images downloaded from the Internet. To simulate this scenario, we distort images from the ETH3D dataset. For each pair of images, we sample undistortion parameters from a distribution $\mathcal {U}$. We detect features on synthetically distorted images using SuperPoint (DeTone et al., 2018), and match them using LightGlue (Lindenberger et al., 2023). The same setup for generating point correspondences given pairs of distorted images applies to Scenario B and C discussed in Sec. 4.5.2 and Sec. 4.5.3, respectively. We apply either the same or different distortions based on the studied setup ($\textbf{F} \lambda$ or $\textbf{F} \lambda _1\lambda _2$). We define $\mathcal {U}$ as a piecewise distribution, which is uniform between $-1.5$ and 0, while its density decreases linearly from $-1.5$ to $-1.8$, reaching half the density of the uniform range. This is done to simulate that in practice, undistortion parameters in the range $[-1.5, 0]$ are more common than in the range $[-1.8, -1.5]$. Thus, it is natural to sample undistortion parameters from a wide range of parameters for the sampling-based approach. We evaluated the sampling-based solvers with $\textbf{U}_1 = \textbf{U}_2 = \{0,-0.6,-1.2\}$. Tab. 1 shows the results for the ETH3D dataset. The Refinement column in the provided tables indicates which parameters are optimized inside LO. We show results for cameras with shared intrinsics ($\lambda _1 = \lambda _2)$ and for two different cameras ($\lambda _1 \not = \lambda _2)$. In this scenario, we also tested variants in which we optimize different distortions even for cameras with the same distortion. As can be seen in Tab. 1, the 6pt $\textbf{E}f$ solver with the sampling-based strategy with $\textbf{U}_1 = \textbf{U}_2 = \{0,-0.6,-1.2\}$ outperforms the dedicated minimal radial distortion solvers that are applied in the first step of RANSAC and the solvers used in combination with GeoCalib for the case of two equal cameras. For the case of two different cameras the best results are obtained by utilizing GeoCalib predictions with the 5pt $\textbf{E}$ solver.

#### Scenario B - *Small distortion*

In the second scenario, we simulate prior knowledge that our cameras have small distortion, *e.g.*, we are processing images taken by mobile phone or DSLR cameras. To simulate this scenario, we distort the feature points with distortions corresponding to undistortion parameters uniformly sampled from the interval $[-0.3, 0]$. In this case, it makes sense to run the 6pt $\textbf{E}f$ and the 7pt $\textbf{F}$ solver in the sampling-based strategy only once with a small undistortion parameter. We decided to use $\textbf{U}_1 = \textbf{U}_2 = \{0\}$ to simulate the standard baseline. Tab. 2 shows the results for this scenario. For both cases the proposed sampling-based and learning-based prior strategies perform significantly better than the dedicated radial distortion solvers. Considering this scenario with shared intrinsics ($\lambda _1 = \lambda _2$), it can be seen that the baseline 6pt $\textbf{E}f$ solver used in combination with the sampling strategy or the GeoCalib predictions performs the best. Similar results can be observed for the case of two different cameras ($\lambda _1 \ne \lambda _2$), where the strategy utilizing GeoCalib provides slightly better accuracy. Here we note, that the overall runtime of the sampling strategy is significantly lower since it does not require a costly neural network inference.

**Table 1:** Prior knowledge about cameras: results on all scenes of the ETH3D dataset, using Poselib RANSAC for synthetic scenario A - *Wild* (*cf.*Sec. 4.5.1). The table shows the average and median pose error in degrees; the Area Under Recall Curve (AUC) at 10$^\circ$; the average and median absolute error $\epsilon (\lambda )$ of the undistortion parameter; the average and median focal length error $\xi (f)$ and the average runtime of RANSAC including the time required for GeoCalib inference for the methods that use it. For the methods using GeoCalib we also provide the runtime of RANSAC in parenthesis. We highlight the bold and bold and italic results

				Poselib - ETH3D- Synth A							
	Minimal	Refinement	Sample	AVG $(^\circ )$ $\downarrow$	MED $(^\circ )$ $\downarrow$	AUC@10 $\uparrow$	AVG $\epsilon (\lambda )$ $\downarrow$	MED $\epsilon (\lambda )$ $\downarrow$	AVG $\xi (f)$ $\downarrow$	MED $\xi (f)$ $\downarrow$	Time (ms) $\downarrow$
$\lambda _1 = \lambda _2$	9pt $\textbf{F} \lambda _1\lambda _2$	$\textbf{R},\vec {t},f,\lambda$	✗	42.46	7.42	0.40	0.47	0.13	0.36	0.21	826
	10pt $\textbf{F} \lambda _1\lambda _2$	$\textbf{R},\vec {t},f,\lambda$	✗	38.45	5.87	0.44	0.41	0.11	0.29	0.19	130
	8pt $\textbf{F} \lambda$	$\textbf{R},\vec {t},f,\lambda$	✗	31.78	2.87	0.56	0.38	0.06	0.34	0.09	579
	9pt $\textbf{F} \lambda$	$\textbf{R},\vec {t},f,\lambda$	✗	31.35	2.97	0.56	0.43	0.07	0.32	0.10	219
	7pt $\textbf{F}$	$\textbf{R},\vec {t},f$	$\lambda$ = 0	50.51	23.74	0.15	0.86	0.87	0.77	0.54	***92***
	7pt $\textbf{F}$	$\textbf{R},\vec {t},f,\lambda$	$\lambda = 0$	43.13	7.41	0.40	0.40	0.12	0.33	0.23	**89**
	7pt $\textbf{F}$	$\textbf{R},\vec {t},f,\lambda$	$\lambda \in \{0.0, -0.6, -1.2\}$	35.36	5.66	0.45	0.29	0.11	0.31	0.19	136
	6pt $\textbf{E}f$	$\textbf{R},\vec {t},f,\lambda$	$\lambda = 0$	15.13	2.05	0.65	0.21	0.05	0.34	0.06	104
	6pt $\textbf{E}f$	$\textbf{R},\vec {t},f,\lambda$	$\lambda \in \{0.0, -0.6, -1.2\}$	**13.86**	**1.76**	**0.68**	0.15	***0.05***	0.32	**0.05**	143
	7pt $\textbf{F}$	$\textbf{R},\vec {t},f$	GeoCalib - $\lambda$	36.23	8.74	0.35	0.24	0.13	0.39	0.28	(82)452
	7pt $\textbf{F}$	$\textbf{R},\vec {t},f,\lambda$	GeoCalib - $\lambda$	35.60	5.95	0.43	0.29	0.10	0.32	0.19	(91)461
	6pt $\textbf{E}f$	$\textbf{R},\vec {t},f$	GeoCalib - $\lambda$	15.63	2.49	0.63	***0.14***	0.06	0.37	0.07	(96)466
	6pt $\textbf{E}f$	$\textbf{R},\vec {t},f,\lambda$	GeoCalib - $\lambda$	***14.45***	***1.96***	***0.67***	**0.13**	**0.04**	0.34	***0.05***	(102)472
	5pt $\textbf{E}$	$\textbf{R}, \vec {t}$	GeoCalib - $\lambda ,f$	19.01	3.87	0.54	0.20	0.10	**0.19**	0.13	(43)413
	5pt $\textbf{E}$	$\textbf{R},\vec {t},f,\lambda$	GeoCalib - $\lambda ,f$	18.70	3.52	0.56	0.23	0.09	0.22	0.13	(115)485
	3pt $\textbf{E}$	$\textbf{R},\vec {t}$	GeoCalib - $\lambda ,f,\vec {g}$	22.12	4.09	0.52	0.20	0.10	***0.19***	0.13	(41)411
	3pt $\textbf{E}$	$\textbf{R},\vec {t},f,\lambda$	GeoCalib - $\lambda ,f,\vec {g}$	25.00	4.32	0.51	0.26	0.10	0.24	0.15	(120)490
$\lambda _1 \ne \lambda _2$	9pt $\textbf{F} \lambda _1\lambda _2$	$\textbf{R},\vec {t},f_1,f_2,\lambda _1,\lambda _2$	✗	46.27	8.80	0.38	0.47	0.13	0.35	0.24	744
	10pt $\textbf{F} \lambda _1\lambda _2$	$\textbf{R},\vec {t},f_1,f_2,\lambda _1, \lambda _2$	✗	37.75	6.07	0.43	0.40	0.10	0.28	0.18	132
	7pt $\textbf{F}$	$\textbf{R},\vec {t},f_1, f_2$	$\lambda _1 = \lambda _2$ = 0	54.77	29.09	0.10	0.88	0.87	0.77	0.60	***102***
	7pt $\textbf{F}$	$\textbf{R},\vec {t},f_1, f_2, \lambda _1, \lambda _2$	$\lambda _1 = \lambda _2 = 0$	46.21	8.79	0.37	0.40	0.12	0.35	0.24	**94**
	7pt $\textbf{F}$	$\textbf{R},\vec {t},f_1, f_2, \lambda _1, \lambda _2$	$\lambda _1, \lambda _2 \in \{0.0, -0.6, -1.2\}$	***32.97***	6.30	0.42	0.27	0.10	0.33	0.20	139
	7pt $\textbf{F}$	$\textbf{R},\vec {t},f_1, f_2$	GeoCalib - $\lambda _1, \lambda _2$	37.74	10.60	0.30	0.30	0.19	0.42	0.31	(84)454
	7pt $\textbf{F}$	$\textbf{R},\vec {t},f_1,f_2,\lambda _1, \lambda _2$	GeoCalib - $\lambda _1, \lambda _2$	35.89	6.62	0.42	0.27	***0.10***	0.32	0.20	(95)465
	5pt $\textbf{E}$	$\textbf{R}, \vec {t}$	GeoCalib - $\lambda _1, \lambda _2,f_1, f_2$	33.15	8.96	0.33	***0.23***	0.16	***0.24***	0.19	(56)426
	5pt $\textbf{E}$	$\textbf{R},\vec {t},f_1, f_2,\lambda _1, \lambda _2$	GeoCalib - $\lambda _1,\lambda _2,f_1,f_2$	**29.44**	**4.75**	**0.49**	**0.23**	**0.09**	**0.24**	**0.16**	(123)493
	3pt $\textbf{E}$	$\textbf{R},\vec {t}$	GeoCalib - $\lambda _1, \lambda _2,f_1, f_2,\vec {g}_1,\vec {g}_2$	34.98	9.18	0.32	0.23	0.16	0.24	0.19	(43)413
	3pt $\textbf{E}$	$\textbf{R},\vec {t},f_1,f_2, \lambda _1, \lambda _2$	GeoCalib - $\lambda _1, \lambda _2, f_1, f_2, \vec {g}_1, \vec {g}_2$	35.16	***5.18***	***0.47***	0.24	0.10	0.24	***0.16***	(123)493

**Table 2:** Prior knowledge about cameras: results on all scenes of the ETH3D dataset, using Poselib RANSAC for synthetic scenario B - *Small Distortion* (*cf.*Sec. 4.5.2). The reported statistics are the same as in Tab. 1

				Poselib - ETH3D- Synth B							
	Minimal	Refinement	Sample	AVG $(^\circ )$ $\downarrow$	MED $(^\circ )$ $\downarrow$	AUC@10 $\uparrow$	AVG $\epsilon (\lambda )$ $\downarrow$	MED $\epsilon (\lambda )$ $\downarrow$	AVG $\xi (f)$ $\downarrow$	MED $\xi (f)$ $\downarrow$	Time (ms) $\downarrow$
$\lambda _1 = \lambda _2$	9pt $\textbf{F} \lambda _1\lambda _2$	$\textbf{R},\vec {t},f,\lambda$	✗	34.15	5.60	0.45	0.27	0.07	0.34	0.16	825
	10pt $\textbf{F} \lambda _1\lambda _2$	$\textbf{R},\vec {t},f,\lambda$	✗	35.87	5.58	0.46	0.31	0.08	0.33	0.17	128
	8pt $\textbf{F} \lambda$	$\textbf{R},\vec {t},f,\lambda$	✗	29.49	2.24	0.59	0.29	0.04	0.36	0.06	650
	9pt $\textbf{F} \lambda$	$\textbf{R},\vec {t},f,\lambda$	✗	29.47	2.28	0.60	0.32	0.04	0.34	0.07	229
	7pt $\textbf{F}$	$\textbf{R},\vec {t},f$	$\lambda$ = 0	33.92	7.43	0.38	0.15	0.15	0.47	0.23	**81**
	7pt $\textbf{F}$	$\textbf{R},\vec {t},f,\lambda$	$\lambda = 0$	32.36	4.90	0.47	0.21	0.07	0.32	0.17	***83***
	6pt $\textbf{E}f$	$\textbf{R},\vec {t},f,\lambda$	$\lambda = 0$	**12.26**	**1.46**	***0.72***	0.09	***0.03***	0.29	**0.04**	95
	7pt $\textbf{F}$	$\textbf{R},\vec {t},f$	GeoCalib - $\lambda$	31.37	5.11	0.47	0.08	0.05	0.37	0.19	(76)446
	7pt $\textbf{F}$	$\textbf{R},\vec {t},f,\lambda$	GeoCalib - $\lambda$	31.28	4.90	0.47	0.19	0.06	0.33	0.16	(82)452
	6pt $\textbf{E}f$	$\textbf{R},\vec {t},f$	GeoCalib - $\lambda$	***12.67***	1.58	0.71	0.06	0.04	0.32	0.04	(91)461
	6pt $\textbf{E}f$	$\textbf{R},\vec {t},f,\lambda$	GeoCalib - $\lambda$	13.04	***1.47***	**0.72**	0.09	**0.03**	0.32	***0.04***	(94)464
	5pt $\textbf{E}$	$\textbf{R}, \vec {t}$	GeoCalib - $\lambda ,f$	23.11	3.93	0.53	**0.05**	0.04	**0.25**	0.17	(40)410
	5pt $\textbf{E}$	$\textbf{R},\vec {t},f,\lambda$	GeoCalib - $\lambda ,f$	22.53	3.81	0.54	0.17	0.07	0.28	0.14	(109)479
	3pt $\textbf{E}$	$\textbf{R},\vec {t}$	GeoCalib - $\lambda ,f,\vec {g}$	27.72	4.32	0.50	***0.05***	0.04	***0.25***	0.17	(37)407
	3pt $\textbf{E}$	$\textbf{R},\vec {t},f,\lambda$	GeoCalib - $\lambda ,f,\vec {g}$	28.56	4.29	0.51	0.21	0.07	0.30	0.15	(115)485
$\lambda _1 \ne \lambda _2$	9pt $\textbf{F} \lambda _1\lambda _2$	$\textbf{R},\vec {t},f_1,f_2,\lambda _1,\lambda _2$	✗	36.79	5.17	0.47	0.30	0.07	0.30	0.16	748
	10pt $\textbf{F} \lambda _1\lambda _2$	$\textbf{R},\vec {t},f_1,f_2,\lambda _1, \lambda _2$	✗	34.73	4.95	0.48	0.32	0.07	**0.29**	**0.15**	130
	7pt $\textbf{F}$	$\textbf{R},\vec {t},f_1, f_2$	$\lambda _1 = \lambda _2$ = 0	34.63	9.14	0.34	0.15	0.15	0.49	0.26	**83**
	7pt $\textbf{F}$	$\textbf{R},\vec {t},f_1, f_2, \lambda _1, \lambda _2$	$\lambda _1 = \lambda _2 = 0$	32.01	4.86	0.48	0.20	0.07	0.31	0.16	***85***
	7pt $\textbf{F}$	$\textbf{R},\vec {t},f_1, f_2$	GeoCalib - $\lambda _1, \lambda _2$	***31.50***	5.83	0.44	0.09	0.06	0.37	0.21	(77)447
	7pt $\textbf{F}$	$\textbf{R},\vec {t},f_1,f_2,\lambda _1, \lambda _2$	GeoCalib - $\lambda _1, \lambda _2$	**30.54**	**4.51**	**0.48**	0.18	0.06	0.30	0.16	(86)456
	5pt $\textbf{E}$	$\textbf{R}, \vec {t}$	GeoCalib - $\lambda _1, \lambda _2,f_1, f_2$	36.26	8.63	0.34	**0.07**	**0.06**	0.30	0.22	(50)420
	5pt $\textbf{E}$	$\textbf{R},\vec {t},f_1, f_2,\lambda _1, \lambda _2$	GeoCalib - $\lambda _1,\lambda _2,f_1,f_2$	33.22	***4.72***	***0.48***	0.17	0.06	***0.29***	***0.15***	(117)487
	3pt $\textbf{E}$	$\textbf{R},\vec {t}$	GeoCalib - $\lambda _1, \lambda _2,f_1, f_2,\vec {g}_1,\vec {g}_2$	38.66	8.86	0.34	***0.07***	***0.06***	0.30	0.22	(42)412
	3pt $\textbf{E}$	$\textbf{R},\vec {t},f_1,f_2, \lambda _1, \lambda _2$	GeoCalib - $\lambda _1, \lambda _2, f_1, f_2, \vec {g}_1, \vec {g}_2$	37.82	5.21	0.46	0.19	0.07	0.30	0.17	(120)490

**Table 3:** Prior knowledge about cameras: results on all scenes of the ETH3D dataset, using Poselib RANSAC for synthetic scenario C - *Visible distortion* (*cf.*Sec. 4.5.3). The reported statistics are the same as in Tab. 1

				Poselib - ETH3D- Synth C							
	Minimal	Refinement	Sample	AVG $(^\circ )$ $\downarrow$	MED $(^\circ )$ $\downarrow$	AUC@10 $\uparrow$	AVG $\epsilon (\lambda )$ $\downarrow$	MED $\epsilon (\lambda )$ $\downarrow$	AVG $\xi (f)$ $\downarrow$	MED $\xi (f)$ $\downarrow$	Time (ms) $\downarrow$
$\lambda _1 = \lambda _2$	9pt $\textbf{F} \lambda _1\lambda _2$	$\textbf{R},\vec {t},f,\lambda$	✗	45.09	8.93	0.37	0.52	0.14	0.36	0.24	759
	10pt $\textbf{F} \lambda _1\lambda _2$	$\textbf{R},\vec {t},f,\lambda$	✗	37.67	6.14	0.43	0.45	0.12	0.29	0.19	129
	8pt $\textbf{F} \lambda$	$\textbf{R},\vec {t},f,\lambda$	✗	31.36	2.97	0.56	0.38	0.07	0.35	0.10	560
	9pt $\textbf{F} \lambda$	$\textbf{R},\vec {t},f,\lambda$	✗	32.45	3.03	0.55	0.43	0.07	0.31	0.11	213
	7pt $\textbf{F}$	$\textbf{R},\vec {t},f$	$\lambda$ = 0	39.03	10.00	0.32	0.29	0.17	0.41	0.31	**82**
	7pt $\textbf{F}$	$\textbf{R},\vec {t},f,\lambda$	$\lambda = -0.9$	38.04	6.48	0.42	0.32	0.11	0.32	0.21	***93***
	7pt $\textbf{F}$	$\textbf{R},\vec {t},f,\lambda$	$\lambda \in \{-0.6, -0.9, -1.2\}$	32.98	5.93	0.44	0.30	0.10	0.33	0.21	136
	6pt $\textbf{E}f$	$\textbf{R},\vec {t},f,\lambda$	$\lambda = -0.9$	**13.04**	***1.83***	***0.69***	0.15	0.05	0.29	0.05	106
	6pt $\textbf{E}f$	$\textbf{R},\vec {t},f,\lambda$	$\lambda \in \{-0.6, -0.9, -1.2\}$	***13.08***	**1.81**	**0.69**	***0.15***	***0.05***	0.32	**0.05**	140
	7pt $\textbf{F}$	$\textbf{R},\vec {t},f$	GeoCalib - $\lambda$	36.87	9.57	0.32	0.30	0.17	0.42	0.31	(81)451
	7pt $\textbf{F}$	$\textbf{R},\vec {t},f,\lambda$	GeoCalib - $\lambda$	36.67	6.89	0.41	0.31	0.11	0.33	0.21	(92)462
	6pt $\textbf{E}f$	$\textbf{R},\vec {t},f$	GeoCalib - $\lambda$	14.80	2.88	0.60	0.17	0.08	0.38	0.09	(96)466
	6pt $\textbf{E}f$	$\textbf{R},\vec {t},f,\lambda$	GeoCalib - $\lambda$	13.27	1.87	0.68	**0.14**	**0.05**	0.34	***0.05***	(103)473
	5pt $\textbf{E}$	$\textbf{R}, \vec {t}$	GeoCalib - $\lambda ,f$	15.54	3.61	0.57	0.25	0.16	**0.17**	0.12	(42)412
	5pt $\textbf{E}$	$\textbf{R},\vec {t},f,\lambda$	GeoCalib - $\lambda ,f$	16.10	3.41	0.57	0.25	0.09	0.20	0.14	(115)485
	3pt $\textbf{E}$	$\textbf{R},\vec {t}$	GeoCalib - $\lambda ,f,\vec {g}$	19.84	3.72	0.55	0.25	0.16	***0.17***	0.12	(41)411
	3pt $\textbf{E}$	$\textbf{R},\vec {t},f,\lambda$	GeoCalib - $\lambda ,f,\vec {g}$	25.00	3.92	0.53	0.27	0.11	0.22	0.14	(121)491
$\lambda _1 \ne \lambda _2$	9pt $\textbf{F} \lambda _1\lambda _2$	$\textbf{R},\vec {t},f_1,f_2,\lambda _1,\lambda _2$	✗	45.39	8.91	0.38	0.49	0.13	0.34	0.23	733
	10pt $\textbf{F} \lambda _1\lambda _2$	$\textbf{R},\vec {t},f_1,f_2,\lambda _1, \lambda _2$	✗	40.36	6.47	0.42	0.44	0.11	0.28	0.19	131
	7pt $\textbf{F}$	$\textbf{R},\vec {t},f_1, f_2$	$\lambda _1 = \lambda _2$ = 0	41.09	12.78	0.25	0.38	0.26	0.46	0.35	**86**
	7pt $\textbf{F}$	$\textbf{R},\vec {t},f_1, f_2, \lambda _1, \lambda _2$	$\lambda _1 = \lambda _2 = -0.9$	37.97	6.66	0.41	0.30	0.11	0.32	0.21	***94***
	7pt $\textbf{F}$	$\textbf{R},\vec {t},f_1, f_2, \lambda _1, \lambda _2$	$\lambda _1, \lambda _2 \in \{-0.6, -0.9, -1.2\}$	34.19	6.14	0.43	0.28	***0.10***	0.32	0.21	137
	7pt $\textbf{F}$	$\textbf{R},\vec {t},f_1, f_2$	GeoCalib - $\lambda _1, \lambda _2$	39.49	12.26	0.26	0.37	0.25	0.44	0.35	(85)455
	7pt $\textbf{F}$	$\textbf{R},\vec {t},f_1,f_2,\lambda _1, \lambda _2$	GeoCalib - $\lambda _1, \lambda _2$	37.28	6.82	0.41	0.30	0.10	0.31	0.21	(94)464
	5pt $\textbf{E}$	$\textbf{R}, \vec {t}$	GeoCalib - $\lambda _1, \lambda _2,f_1, f_2$	***26.74***	7.93	0.36	***0.27***	0.22	**0.21**	0.17	(51)421
	5pt $\textbf{E}$	$\textbf{R},\vec {t},f_1, f_2,\lambda _1, \lambda _2$	GeoCalib - $\lambda _1,\lambda _2,f_1,f_2$	**23.89**	**4.38**	**0.51**	**0.25**	**0.10**	0.21	***0.15***	(120)490
	3pt $\textbf{E}$	$\textbf{R},\vec {t}$	GeoCalib - $\lambda _1, \lambda _2,f_1, f_2,\vec {g}_1,\vec {g}_2$	29.35	8.14	0.35	0.27	0.22	***0.21***	0.17	(43)413
	3pt $\textbf{E}$	$\textbf{R},\vec {t},f_1,f_2, \lambda _1, \lambda _2$	GeoCalib - $\lambda _1, \lambda _2, f_1, f_2, \vec {g}_1, \vec {g}_2$	29.78	***4.90***	***0.48***	0.29	0.11	0.23	**0.15**	(122)492

**Table 4:** Natural scenes: results on all scenes of the PragueParks dataset, using Poselib RANSAC for synthetic scenario A - *Wild* (*cf.*Sec. 4.5.1). The reported statistics are the same as in Tab. 1

				Poselib - Prague Parks - Synth A							
	Minimal	Refinement	Sample	AVG $(^\circ )$ $\downarrow$	MED $(^\circ )$ $\downarrow$	AUC@10 $\uparrow$	AVG $\epsilon (\lambda )$ $\downarrow$	MED $\epsilon (\lambda )$ $\downarrow$	AVG $\xi (f)$ $\downarrow$	MED $\xi (f)$ $\downarrow$	Time (ms) $\downarrow$
$\lambda _1 = \lambda _2$	9pt $\textbf{F} \lambda _1\lambda _2$	$\textbf{R},\vec {t},f,\lambda$	✗	21.47	4.02	0.55	0.15	0.08	0.21	0.12	360
	10pt $\textbf{F} \lambda _1\lambda _2$	$\textbf{R},\vec {t},f,\lambda$	✗	15.40	3.75	0.59	0.14	0.08	0.19	0.11	117
	8pt $\textbf{F} \lambda$	$\textbf{R},\vec {t},f,\lambda$	✗	10.92	1.82	0.74	0.12	***0.06***	**0.16**	0.07	282
	9pt $\textbf{F} \lambda$	$\textbf{R},\vec {t},f,\lambda$	✗	11.60	1.80	0.72	0.13	0.06	***0.19***	0.07	144
	7pt $\textbf{F}$	$\textbf{R},\vec {t},f$	$\lambda$ = 0	33.25	19.66	0.15	0.87	0.89	0.87	0.66	116
	7pt $\textbf{F}$	$\textbf{R},\vec {t},f,\lambda$	$\lambda = 0$	18.55	4.11	0.56	0.17	0.09	0.24	0.13	**97**
	7pt $\textbf{F}$	$\textbf{R},\vec {t},f,\lambda$	$\lambda \in \{0.0, -0.6, -1.2\}$	13.29	3.41	0.60	0.13	0.08	0.22	0.12	136
	6pt $\textbf{E}f$	$\textbf{R},\vec {t},f,\lambda$	$\lambda = 0$	11.48	1.82	0.71	0.13	0.07	0.24	0.07	***110***
	6pt $\textbf{E}f$	$\textbf{R},\vec {t},f,\lambda$	$\lambda \in \{0.0, -0.6, -1.2\}$	**7.45**	**1.58**	**0.76**	**0.10**	**0.06**	0.21	**0.07**	140
	7pt $\textbf{F}$	$\textbf{R},\vec {t},f$	GeoCalib - $\lambda$	20.48	6.82	0.39	0.34	0.19	0.38	0.22	(98)468
	7pt $\textbf{F}$	$\textbf{R},\vec {t},f,\lambda$	GeoCalib - $\lambda$	16.12	3.68	0.59	0.13	0.08	0.21	0.12	(97)467
	6pt $\textbf{E}f$	$\textbf{R},\vec {t},f$	GeoCalib - $\lambda$	13.86	2.80	0.63	0.26	0.11	0.37	0.10	(116)486
	6pt $\textbf{E}f$	$\textbf{R},\vec {t},f,\lambda$	GeoCalib - $\lambda$	***9.20***	***1.79***	***0.74***	***0.11***	0.06	0.19	***0.07***	(108)478
	5pt $\textbf{E}$	$\textbf{R}, \vec {t}$	GeoCalib - $\lambda ,f$	24.39	8.17	0.32	0.45	0.27	0.70	0.61	(56)426
	5pt $\textbf{E}$	$\textbf{R},\vec {t},f,\lambda$	GeoCalib - $\lambda ,f$	21.36	7.22	0.40	0.17	0.11	0.47	0.31	(132)502
	3pt $\textbf{E}$	$\textbf{R},\vec {t}$	GeoCalib - $\lambda ,f,\vec {g}$	42.89	13.67	0.24	0.45	0.27	0.70	0.61	(52)422
	3pt $\textbf{E}$	$\textbf{R},\vec {t},f,\lambda$	GeoCalib - $\lambda ,f,\vec {g}$	46.57	12.18	0.28	0.26	0.15	0.58	0.43	(156)526
$\lambda _1 \ne \lambda _2$	9pt $\textbf{F} \lambda _1\lambda _2$	$\textbf{R},\vec {t},f_1,f_2,\lambda _1,\lambda _2$	✗	26.04	4.50	0.50	0.15	**0.08**	0.23	0.14	448
	10pt $\textbf{F} \lambda _1\lambda _2$	$\textbf{R},\vec {t},f_1,f_2,\lambda _1, \lambda _2$	✗	***14.76***	**3.75**	**0.58**	**0.13**	***0.08***	**0.18**	**0.12**	***136***
	7pt $\textbf{F}$	$\textbf{R},\vec {t},f_1,f_2$	$\lambda _1 = \lambda _2$ = 0	42.77	29.66	0.07	0.92	0.94	0.85	0.70	169
	7pt $\textbf{F}$	$\textbf{R},\vec {t},f_1,f_2, \lambda _1, \lambda _2$	$\lambda _1 = \lambda _2 = 0$	23.48	4.54	0.50	0.20	0.09	0.23	0.14	**113**
	7pt $\textbf{F}$	$\textbf{R},\vec {t},f_1,f_2, \lambda _1, \lambda _2$	$\lambda _1, \lambda _2 \in \{0.0, -0.6, -1.2\}$	**13.73**	***3.85***	***0.55***	***0.13***	0.09	***0.22***	0.13	146
	7pt $\textbf{F}$	$\textbf{R},\vec {t},f_1,f_2$	GeoCalib - $\lambda _1, \lambda _2$	29.44	12.48	0.22	0.53	0.43	0.50	0.39	(143)513
	7pt $\textbf{F}$	$\textbf{R},\vec {t},f_1,f_2,\lambda _1, \lambda _2$	GeoCalib - $\lambda _1, \lambda _2$	20.00	4.13	0.53	0.15	0.09	0.24	***0.13***	(115)485
	5pt $\textbf{E}$	$\textbf{R}, \vec {t}$	GeoCalib - $\lambda _1, \lambda _2,f_1, f_2$	50.31	24.73	0.11	0.51	0.46	0.73	0.63	(116)486
	5pt $\textbf{E}$	$\textbf{R},\vec {t},f_1, f_2,\lambda _1, \lambda _2$	GeoCalib - $\lambda _1,\lambda _2,f_1,f_2$	44.49	12.68	0.25	0.23	0.16	0.58	0.46	(180)550
	3pt $\textbf{E}$	$\textbf{R},\vec {t}$	GeoCalib - $\lambda _1, \lambda _2,f_1, f_2,\vec {g}_1,\vec {g}_2$	54.64	29.88	0.09	0.51	0.46	0.73	0.63	(58)428
	3pt $\textbf{E}$	$\textbf{R},\vec {t},f_1,f_2, \lambda _1, \lambda _2$	GeoCalib - $\lambda _1, \lambda _2, f_1, f_2, \vec {g}_1, \vec {g}_2$	54.16	17.86	0.21	0.30	0.18	0.63	0.53	(168)538

**Table 5:** Natural scenes: results on all scenes of the PragueParks dataset, using Poselib RANSAC for synthetic scenario B - *Small Distortion* (*cf.*Sec. 4.5.2). The reported statistics are the same as in Tab. 1

				Poselib - Prague Parks - Synth B							
	Minimal	Refinement	Sample	AVG $(^\circ )$ $\downarrow$	MED $(^\circ )$ $\downarrow$	AUC@10 $\uparrow$	AVG $\epsilon (\lambda )$ $\downarrow$	MED $\epsilon (\lambda )$ $\downarrow$	AVG $\xi (f)$ $\downarrow$	MED $\xi (f)$ $\downarrow$	Time (ms) $\downarrow$
$\lambda _1 = \lambda _2$	9pt $\textbf{F} \lambda _1\lambda _2$	$\textbf{R},\vec {t},f,\lambda$	✗	14.10	2.78	0.65	0.08	0.05	0.18	0.09	309
	10pt $\textbf{F} \lambda _1\lambda _2$	$\textbf{R},\vec {t},f,\lambda$	✗	11.27	2.64	0.66	0.08	0.05	0.17	0.08	102
	8pt $\textbf{F} \lambda$	$\textbf{R},\vec {t},f,\lambda$	✗	9.91	1.40	0.78	0.07	0.04	0.15	0.04	247
	9pt $\textbf{F} \lambda$	$\textbf{R},\vec {t},f,\lambda$	✗	7.40	1.44	0.79	0.08	0.04	**0.11**	0.05	126
	7pt $\textbf{F}$	$\textbf{R},\vec {t},f$	$\lambda$ = 0	14.05	5.06	0.49	0.15	0.15	0.34	0.19	**80**
	7pt $\textbf{F}$	$\textbf{R},\vec {t},f,\lambda$	$\lambda = 0$	12.01	2.65	0.66	0.08	0.06	0.17	0.08	***80***
	6pt $\textbf{E}f$	$\textbf{R},\vec {t},f,\lambda$	$\lambda = 0$	***6.18***	***1.38***	***0.81***	**0.06**	***0.04***	0.13	***0.04***	90
	7pt $\textbf{F}$	$\textbf{R},\vec {t},f$	GeoCalib - $\lambda$	13.15	4.04	0.53	0.12	0.08	0.28	0.12	(84)454
	7pt $\textbf{F}$	$\textbf{R},\vec {t},f,\lambda$	GeoCalib - $\lambda$	9.63	2.73	0.66	0.08	0.05	0.17	0.09	(83)453
	6pt $\textbf{E}f$	$\textbf{R},\vec {t},f$	GeoCalib - $\lambda$	7.06	2.14	0.71	0.12	0.08	0.18	0.08	(97)467
	6pt $\textbf{E}f$	$\textbf{R},\vec {t},f,\lambda$	GeoCalib - $\lambda$	**5.56**	**1.35**	**0.81**	***0.06***	**0.04**	***0.13***	**0.04**	(95)465
	5pt $\textbf{E}$	$\textbf{R}, \vec {t}$	GeoCalib - $\lambda ,f$	23.73	6.98	0.40	0.23	0.15	0.50	0.42	(50)420
	5pt $\textbf{E}$	$\textbf{R},\vec {t},f,\lambda$	GeoCalib - $\lambda ,f$	21.87	3.88	0.54	0.09	0.06	0.28	0.12	(119)489
	3pt $\textbf{E}$	$\textbf{R},\vec {t}$	GeoCalib - $\lambda ,f,\vec {g}$	39.63	11.41	0.30	0.23	0.15	0.50	0.42	(45)415
	3pt $\textbf{E}$	$\textbf{R},\vec {t},f,\lambda$	GeoCalib - $\lambda ,f,\vec {g}$	37.50	5.81	0.43	0.14	0.07	0.36	0.16	(134)504
$\lambda _1 \ne \lambda _2$	9pt $\textbf{F} \lambda _1\lambda _2$	$\textbf{R},\vec {t},f_1,f_2,\lambda _1,\lambda _2$	✗	16.16	3.00	***0.64***	***0.08***	***0.06***	0.17	***0.09***	317
	10pt $\textbf{F} \lambda _1\lambda _2$	$\textbf{R},\vec {t},f_1,f_2,\lambda _1, \lambda _2$	✗	***13.65***	3.00	**0.64**	**0.08**	**0.06**	**0.15**	0.09	110
	7pt $\textbf{F}$	$\textbf{R},\vec {t},f_1, f_2$	$\lambda _1 = \lambda _2$ = 0	20.82	8.48	0.34	0.15	0.14	0.52	0.26	***94***
	7pt $\textbf{F}$	$\textbf{R},\vec {t},f_1, f_2, \lambda _1, \lambda _2$	$\lambda _1 = \lambda _2 = 0$	**11.80**	***2.87***	0.63	0.08	0.06	0.18	0.09	**89**
	7pt $\textbf{F}$	$\textbf{R},\vec {t},f_1, f_2$	GeoCalib - $\lambda _1, \lambda _2$	23.80	8.65	0.34	0.16	0.12	0.51	0.25	(107)477
	7pt $\textbf{F}$	$\textbf{R},\vec {t},f_1,f_2,\lambda _1, \lambda _2$	GeoCalib - $\lambda _1, \lambda _2$	14.98	**2.84**	0.64	0.09	0.06	***0.16***	**0.08**	(95)465
	5pt $\textbf{E}$	$\textbf{R}, \vec {t}$	GeoCalib - $\lambda _1, \lambda _2,f_1, f_2$	48.20	20.63	0.16	0.29	0.23	0.59	0.42	(92)462
	5pt $\textbf{E}$	$\textbf{R},\vec {t},f_1, f_2,\lambda _1, \lambda _2$	GeoCalib - $\lambda _1,\lambda _2,f_1,f_2$	41.94	7.36	0.39	0.15	0.07	0.45	0.19	(156)526
	3pt $\textbf{E}$	$\textbf{R},\vec {t}$	GeoCalib - $\lambda _1, \lambda _2,f_1, f_2,\vec {g}_1,\vec {g}_2$	49.12	23.29	0.16	0.29	0.23	0.59	0.42	(53)423
	3pt $\textbf{E}$	$\textbf{R},\vec {t},f_1,f_2, \lambda _1, \lambda _2$	GeoCalib - $\lambda _1, \lambda _2, f_1, f_2, \vec {g}_1, \vec {g}_2$	44.63	9.57	0.36	0.20	0.09	0.50	0.24	(155)525

**Table 6:** Natural scenes: results on all scenes of the PragueParks dataset, using Poselib RANSAC for synthetic scenario C - *Visible distortion* (*cf.*Sec. 4.5.3). The reported statistics are the same as in Tab. 1

				Poselib - Prague Parks - Synth C							
	Minimal	Refinement	Sample	AVG $(^\circ )$ $\downarrow$	MED $(^\circ )$ $\downarrow$	AUC@10 $\uparrow$	AVG $\epsilon (\lambda )$ $\downarrow$	MED $\epsilon (\lambda )$ $\downarrow$	AVG $\xi (f)$ $\downarrow$	MED $\xi (f)$ $\downarrow$	Time (ms) $\downarrow$
$\lambda _1 = \lambda _2$	9pt $\textbf{F} \lambda _1\lambda _2$	$\textbf{R},\vec {t},f,\lambda$	✗	22.52	4.29	0.53	0.17	0.11	0.23	0.14	417
	10pt $\textbf{F} \lambda _1\lambda _2$	$\textbf{R},\vec {t},f,\lambda$	✗	15.08	3.62	0.59	0.15	0.09	***0.19***	0.12	127
	8pt $\textbf{F} \lambda$	$\textbf{R},\vec {t},f,\lambda$	✗	12.98	1.94	0.71	0.14	**0.07**	**0.18**	0.07	259
	9pt $\textbf{F} \lambda$	$\textbf{R},\vec {t},f,\lambda$	✗	11.82	2.01	0.70	0.13	0.08	0.19	0.08	141
	7pt $\textbf{F}$	$\textbf{R},\vec {t},f$	$\lambda$ = 0	16.68	6.72	0.40	0.23	0.15	0.35	0.21	**99**
	7pt $\textbf{F}$	$\textbf{R},\vec {t},f,\lambda$	$\lambda = -0.9$	13.60	3.82	0.57	0.14	0.09	0.23	0.13	***102***
	7pt $\textbf{F}$	$\textbf{R},\vec {t},f,\lambda$	$\lambda \in \{-0.6, -0.9, -1.2\}$	11.19	3.35	0.60	0.14	0.09	0.22	0.12	139
	6pt $\textbf{E}f$	$\textbf{R},\vec {t},f,\lambda$	$\lambda = -0.9$	***7.97***	1.95	***0.71***	***0.11***	0.07	0.23	0.07	112
	6pt $\textbf{E}f$	$\textbf{R},\vec {t},f,\lambda$	$\lambda \in \{-0.6, -0.9, -1.2\}$	**7.46**	***1.89***	**0.73**	**0.11**	***0.07***	0.20	***0.07***	141
	7pt $\textbf{F}$	$\textbf{R},\vec {t},f$	GeoCalib - $\lambda$	22.29	8.09	0.36	0.41	0.22	0.40	0.24	(104)474
	7pt $\textbf{F}$	$\textbf{R},\vec {t},f,\lambda$	GeoCalib - $\lambda$	16.04	3.88	0.57	0.15	0.10	0.22	0.13	(105)475
	6pt $\textbf{E}f$	$\textbf{R},\vec {t},f$	GeoCalib - $\lambda$	14.50	3.18	0.58	0.30	0.13	0.40	0.12	(119)489
	6pt $\textbf{E}f$	$\textbf{R},\vec {t},f,\lambda$	GeoCalib - $\lambda$	11.37	**1.84**	0.71	0.12	0.08	0.21	**0.07**	(111)481
	5pt $\textbf{E}$	$\textbf{R}, \vec {t}$	GeoCalib - $\lambda ,f$	26.75	10.06	0.29	0.52	0.38	0.74	0.68	(59)429
	5pt $\textbf{E}$	$\textbf{R},\vec {t},f,\lambda$	GeoCalib - $\lambda ,f$	23.94	7.51	0.36	0.19	0.14	0.50	0.38	(144)514
	3pt $\textbf{E}$	$\textbf{R},\vec {t}$	GeoCalib - $\lambda ,f,\vec {g}$	44.56	14.44	0.23	0.52	0.38	0.74	0.68	(52)422
	3pt $\textbf{E}$	$\textbf{R},\vec {t},f,\lambda$	GeoCalib - $\lambda ,f,\vec {g}$	49.75	12.96	0.26	0.28	0.17	0.61	0.49	(154)524
$\lambda _1 \ne \lambda _2$	9pt $\textbf{F} \lambda _1\lambda _2$	$\textbf{R},\vec {t},f_1,f_2,\lambda _1,\lambda _2$	✗	25.42	4.61	0.50	0.19	0.10	0.26	0.13	410
	10pt $\textbf{F} \lambda _1\lambda _2$	$\textbf{R},\vec {t},f_1,f_2,\lambda _1, \lambda _2$	✗	***14.86***	**3.52**	**0.59**	0.17	0.09	**0.19**	**0.12**	129
	7pt $\textbf{F}$	$\textbf{R},\vec {t},f_1, f_2$	$\lambda _1 = \lambda _2$ = 0	27.09	11.64	0.25	0.37	0.28	0.46	0.35	***116***
	7pt $\textbf{F}$	$\textbf{R},\vec {t},f_1, f_2, \lambda _1, \lambda _2$	$\lambda _1 = \lambda _2 = -0.9$	19.66	3.88	0.54	0.16	0.09	0.22	0.12	**106**
	7pt $\textbf{F}$	$\textbf{R},\vec {t},f_1, f_2, \lambda _1, \lambda _2$	$\lambda _1, \lambda _2 \in \{-0.6, -0.9, -1.2\}$	**14.05**	***3.59***	***0.57***	**0.14**	***0.09***	0.21	***0.12***	142
	7pt $\textbf{F}$	$\textbf{R},\vec {t},f_1, f_2$	GeoCalib - $\lambda _1, \lambda _2$	31.14	13.03	0.21	0.65	0.60	0.54	0.36	(130)500
	7pt $\textbf{F}$	$\textbf{R},\vec {t},f_1,f_2,\lambda _1, \lambda _2$	GeoCalib - $\lambda _1, \lambda _2$	23.02	3.86	0.55	***0.15***	**0.09**	***0.21***	0.12	(108)478
	5pt $\textbf{E}$	$\textbf{R}, \vec {t}$	GeoCalib - $\lambda _1, \lambda _2,f_1, f_2$	48.61	25.03	0.10	0.57	0.56	0.74	0.68	(97)467
	5pt $\textbf{E}$	$\textbf{R},\vec {t},f_1, f_2,\lambda _1, \lambda _2$	GeoCalib - $\lambda _1,\lambda _2,f_1,f_2$	44.39	13.22	0.25	0.24	0.17	0.59	0.52	(164)534
	3pt $\textbf{E}$	$\textbf{R},\vec {t}$	GeoCalib - $\lambda _1, \lambda _2,f_1, f_2,\vec {g}_1,\vec {g}_2$	56.90	32.67	0.09	0.57	0.56	0.74	0.68	(56)426
	3pt $\textbf{E}$	$\textbf{R},\vec {t},f_1,f_2, \lambda _1, \lambda _2$	GeoCalib - $\lambda _1, \lambda _2, f_1, f_2, \vec {g}_1, \vec {g}_2$	53.21	18.16	0.19	0.34	0.24	0.62	0.56	(166)536

**Table 7:** Results for the ROTUNDA scene using Poselib RANSAC. The reported statistics are the same as in Tab. 1

				Poselib - ROTUNDA							
	Minimal	Refinement	Sample	AVG $(^\circ )$ $\downarrow$	MED $(^\circ )$ $\downarrow$	AUC@10 $\uparrow$	AVG $\epsilon (\lambda )$ $\downarrow$	MED $\epsilon (\lambda )$ $\downarrow$	AVG $\xi (f)$ $\downarrow$	MED $\xi (f)$ $\downarrow$	Time (ms) $\downarrow$
$\lambda _1 = \lambda _2$	9pt $\textbf{F} \lambda _1\lambda _2$	$\textbf{R},\vec {t},f,\lambda$	✗	26.22	8.60	0.35	0.59	0.13	0.25	0.14	3057
	10pt $\textbf{F} \lambda _1\lambda _2$	$\textbf{R},\vec {t},f,\lambda$	✗	26.56	7.68	0.37	0.58	0.12	0.25	0.14	229
	8pt $\textbf{F} \lambda$	$\textbf{R},\vec {t},f,\lambda$	✗	25.81	4.37	0.48	0.49	0.12	0.28	0.08	1945
	9pt $\textbf{F} \lambda$	$\textbf{R},\vec {t},f,\lambda$	✗	26.21	6.09	0.44	0.64	0.14	0.27	0.11	496
	7pt $\textbf{F}$	$\textbf{R},\vec {t},f$	$\lambda$ = 0	26.00	11.66	0.29	0.53	0.09	0.40	0.22	***90***
	7pt $\textbf{F}$	$\textbf{R},\vec {t},f,\lambda$	$\lambda = 0$	23.58	8.45	0.35	0.41	0.12	0.30	0.16	**82**
	7pt $\textbf{F}$	$\textbf{R},\vec {t},f,\lambda$	$\lambda \in \{0.0, -0.6, -1.2\}$	20.94	6.50	0.41	0.27	0.11	0.25	0.13	272
	6pt $\textbf{E}f$	$\textbf{R},\vec {t},f,\lambda$	$\lambda = 0$	19.98	3.78	0.52	0.36	0.10	0.31	0.07	171
	6pt $\textbf{E}f$	$\textbf{R},\vec {t},f,\lambda$	$\lambda \in \{0.0, -0.6, -1.2\}$	***17.80***	***2.83***	***0.58***	0.23	***0.08***	0.26	***0.05***	347
	7pt $\textbf{F}$	$\textbf{R},\vec {t},f$	GeoCalib - $\lambda$	20.77	7.24	0.38	0.17	0.14	0.27	0.14	(76)446
	7pt $\textbf{F}$	$\textbf{R},\vec {t},f,\lambda$	GeoCalib - $\lambda$	19.79	6.26	0.42	0.19	0.09	**0.24**	0.12	(77)447
	6pt $\textbf{E}f$	$\textbf{R},\vec {t},f$	GeoCalib - $\lambda$	17.94	3.47	0.55	***0.16***	0.14	0.28	0.07	(155)525
	6pt $\textbf{E}f$	$\textbf{R},\vec {t},f,\lambda$	GeoCalib - $\lambda$	**17.10**	**2.71**	**0.59**	**0.13**	**0.08**	0.25	**0.05**	(151)521
	5pt $\textbf{E}$	$\textbf{R}, \vec {t}$	GeoCalib - $\lambda ,f$	31.92	8.60	0.35	0.20	0.19	0.32	0.17	(46)416
	5pt $\textbf{E}$	$\textbf{R},\vec {t},f,\lambda$	GeoCalib - $\lambda ,f$	31.20	7.99	0.37	0.21	0.11	***0.24***	0.11	(106)476
	3pt $\textbf{E}$	$\textbf{R},\vec {t}$	GeoCalib - $\lambda ,f,\vec {g}$	46.06	25.60	0.26	0.20	0.19	0.32	0.17	(36)406
	3pt $\textbf{E}$	$\textbf{R},\vec {t},f,\lambda$	GeoCalib - $\lambda ,f,\vec {g}$	47.74	21.25	0.26	0.47	0.25	0.28	0.14	(104)474
$\lambda _1 \ne \lambda _2$	9pt $\textbf{F} \lambda _1\lambda _2$	$\textbf{R},\vec {t},f_1,f_2,\lambda _1,\lambda _2$	✗	40.40	12.64	0.29	1.01	0.25	0.37	0.25	3830
	10pt $\textbf{F} \lambda _1\lambda _2$	$\textbf{R},\vec {t},f_1,f_2,\lambda _1, \lambda _2$	✗	40.16	11.54	***0.31***	1.09	0.25	0.37	0.25	249
	7pt $\textbf{F}$	$\textbf{R},\vec {t},f_1, f_2$	$\lambda _1 = \lambda _2$ = 0	39.74	16.29	0.24	0.55	**0.09**	0.49	0.34	***83***
	7pt $\textbf{F}$	$\textbf{R},\vec {t},f_1, f_2, \lambda _1, \lambda _2$	$\lambda _1 = \lambda _2 = 0$	38.22	12.58	0.29	0.51	0.22	0.39	0.29	**76**
	7pt $\textbf{F}$	$\textbf{R},\vec {t},f_1, f_2, \lambda _1, \lambda _2$	$\lambda _1, \lambda _2 \in \{0.0, -0.6, -1.2\}$	**35.43**	**9.66**	**0.34**	0.39	0.18	0.35	0.26	320
	7pt $\textbf{F}$	$\textbf{R},\vec {t},f_1, f_2$	GeoCalib - $\lambda _1, \lambda _2$	39.12	14.61	0.23	**0.20**	0.17	0.43	0.32	(79)449
	7pt $\textbf{F}$	$\textbf{R},\vec {t},f_1,f_2,\lambda _1, \lambda _2$	GeoCalib - $\lambda _1, \lambda _2$	***36.69***	11.47	0.31	0.30	***0.16***	0.37	0.28	(75)445
	5pt $\textbf{E}$	$\textbf{R}, \vec {t}$	GeoCalib - $\lambda _1, \lambda _2,f_1, f_2$	42.40	15.38	0.24	***0.23***	0.22	0.35	0.23	(60)430
	5pt $\textbf{E}$	$\textbf{R},\vec {t},f_1, f_2,\lambda _1, \lambda _2$	GeoCalib - $\lambda _1,\lambda _2,f_1,f_2$	41.65	***10.80***	0.30	0.27	0.17	**0.29**	**0.18**	(106)476
	3pt $\textbf{E}$	$\textbf{R},\vec {t}$	GeoCalib - $\lambda _1, \lambda _2,f_1, f_2,\vec {g}_1,\vec {g}_2$	53.47	38.82	0.16	0.23	0.22	0.35	0.23	(29)399
	3pt $\textbf{E}$	$\textbf{R},\vec {t},f_1,f_2, \lambda _1, \lambda _2$	GeoCalib - $\lambda _1, \lambda _2, f_1, f_2, \vec {g}_1, \vec {g}_2$	52.26	33.52	0.19	0.57	0.33	***0.33***	***0.21***	(88)458

**Table 8:** Results for the CATHEDRAL scene using Poselib RANSAC. The reported statistics are the same as in Tab. 1

				Poselib - CATHEDRAL							
	Minimal	Refinement	Sample	AVG $(^\circ )$ $\downarrow$	MED $(^\circ )$ $\downarrow$	AUC@10 $\uparrow$	AVG $\epsilon (\lambda )$ $\downarrow$	MED $\epsilon (\lambda )$ $\downarrow$	AVG $\xi (f)$ $\downarrow$	MED $\xi (f)$ $\downarrow$	Time (ms) $\downarrow$
$\lambda _1 = \lambda _2$	9pt $\textbf{F} \lambda _1\lambda _2$	$\textbf{R},\vec {t},f,\lambda$	✗	14.98	2.81	0.60	0.27	0.07	0.24	0.09	1559
	10pt $\textbf{F} \lambda _1\lambda _2$	$\textbf{R},\vec {t},f,\lambda$	✗	13.05	2.51	0.63	0.25	0.06	0.20	0.08	195
	8pt $\textbf{F} \lambda$	$\textbf{R},\vec {t},f,\lambda$	✗	10.16	1.41	0.73	0.23	0.05	0.18	0.05	924
	9pt $\textbf{F} \lambda$	$\textbf{R},\vec {t},f,\lambda$	✗	10.41	1.48	0.72	0.25	0.05	0.19	0.05	318
	7pt $\textbf{F}$	$\textbf{R},\vec {t},f$	$\lambda$ = 0	21.56	9.44	0.37	0.69	0.72	0.56	0.26	***150***
	7pt $\textbf{F}$	$\textbf{R},\vec {t},f,\lambda$	$\lambda = 0$	15.04	2.84	0.60	0.24	0.07	0.25	0.09	**117**
	7pt $\textbf{F}$	$\textbf{R},\vec {t},f,\lambda$	$\lambda \in \{0.0, -0.6, -1.2\}$	11.91	2.34	0.65	0.18	0.06	0.23	0.08	239
	6pt $\textbf{E}f$	$\textbf{R},\vec {t},f,\lambda$	$\lambda = 0$	9.15	1.47	0.72	0.21	0.05	0.25	0.05	161
	6pt $\textbf{E}f$	$\textbf{R},\vec {t},f,\lambda$	$\lambda \in \{0.0, -0.6, -1.2\}$	7.24	**1.23**	***0.77***	0.15	***0.04***	0.20	0.04	251
	7pt $\textbf{F}$	$\textbf{R},\vec {t},f$	GeoCalib - $\lambda$	12.49	2.71	0.62	0.14	0.06	0.25	0.09	(110)480
	7pt $\textbf{F}$	$\textbf{R},\vec {t},f,\lambda$	GeoCalib - $\lambda$	11.68	2.32	0.65	0.16	0.06	0.22	0.08	(114)484
	6pt $\textbf{E}f$	$\textbf{R},\vec {t},f$	GeoCalib - $\lambda$	7.70	1.38	0.76	**0.13**	0.04	0.20	0.04	(149)519
	6pt $\textbf{E}f$	$\textbf{R},\vec {t},f,\lambda$	GeoCalib - $\lambda$	7.18	***1.23***	**0.77**	***0.13***	**0.04**	0.18	0.04	(148)518
	5pt $\textbf{E}$	$\textbf{R}, \vec {t}$	GeoCalib - $\lambda ,f$	**6.48**	1.82	0.74	0.13	0.07	**0.03**	**0.02**	(50)420
	5pt $\textbf{E}$	$\textbf{R},\vec {t},f,\lambda$	GeoCalib - $\lambda ,f$	7.58	2.01	0.71	0.16	0.05	0.15	0.06	(142)512
	3pt $\textbf{E}$	$\textbf{R},\vec {t}$	GeoCalib - $\lambda ,f,\vec {g}$	***6.56***	1.82	0.74	0.13	0.07	***0.03***	***0.02***	(37)407
	3pt $\textbf{E}$	$\textbf{R},\vec {t},f,\lambda$	GeoCalib - $\lambda ,f,\vec {g}$	9.21	2.13	0.69	0.17	0.06	0.16	0.07	(126)496
$\lambda _1 \ne \lambda _2$	9pt $\textbf{F} \lambda _1\lambda _2$	$\textbf{R},\vec {t},f_1,f_2,\lambda _1,\lambda _2$	✗	17.19	4.24	0.51	0.32	0.12	0.32	0.27	2942
	10pt $\textbf{F} \lambda _1\lambda _2$	$\textbf{R},\vec {t},f_1,f_2,\lambda _1, \lambda _2$	✗	15.04	3.74	0.54	0.32	0.10	0.28	0.25	283
	7pt $\textbf{F}$	$\textbf{R},\vec {t},f_1, f_2$	$\lambda _1 = \lambda _2$ = 0	27.93	14.45	0.22	0.64	0.68	0.59	0.39	***184***
	7pt $\textbf{F}$	$\textbf{R},\vec {t},f_1, f_2, \lambda _1, \lambda _2$	$\lambda _1 = \lambda _2 = 0$	20.18	4.60	0.49	0.29	0.12	0.33	0.29	**121**
	7pt $\textbf{F}$	$\textbf{R},\vec {t},f_1, f_2, \lambda _1, \lambda _2$	$\lambda _1, \lambda _2 \in \{0.0, -0.6, -1.2\}$	15.89	3.58	0.55	0.22	0.09	0.31	0.28	310
	7pt $\textbf{F}$	$\textbf{R},\vec {t},f_1, f_2$	GeoCalib - $\lambda _1, \lambda _2$	16.74	4.53	0.50	0.15	**0.08**	0.33	0.28	(121)491
	7pt $\textbf{F}$	$\textbf{R},\vec {t},f_1,f_2,\lambda _1, \lambda _2$	GeoCalib - $\lambda _1, \lambda _2$	15.76	3.53	0.56	0.20	***0.09***	0.30	0.27	(115)485
	5pt $\textbf{E}$	$\textbf{R}, \vec {t}$	GeoCalib - $\lambda _1, \lambda _2,f_1, f_2$	***8.91***	***3.18***	***0.60***	**0.13**	0.09	**0.17**	**0.05**	(60)430
	5pt $\textbf{E}$	$\textbf{R},\vec {t},f_1, f_2,\lambda _1, \lambda _2$	GeoCalib - $\lambda _1,\lambda _2,f_1,f_2$	**8.79**	**2.98**	**0.61**	0.20	0.09	0.24	0.20	(142)512
	3pt $\textbf{E}$	$\textbf{R},\vec {t}$	GeoCalib - $\lambda _1, \lambda _2,f_1, f_2,\vec {g}_1,\vec {g}_2$	9.45	3.23	0.59	***0.13***	0.09	***0.17***	***0.05***	(32)402
	3pt $\textbf{E}$	$\textbf{R},\vec {t},f_1,f_2, \lambda _1, \lambda _2$	GeoCalib - $\lambda _1, \lambda _2, f_1, f_2, \vec {g}_1, \vec {g}_2$	10.16	3.21	0.60	0.22	0.09	0.25	0.21	(109)479

**Table 9:** Results for the EuRoC-MAV dataset using Poselib RANSAC. The reported statistics are the same as in Tab. 1. We do not include statistics for the radial undistortion parameter estimation since GT data is not provided for division model

				Poselib - Euroc							
	Minimal	Refinement	Sample	AVG $(^\circ )$ $\downarrow$	MED $(^\circ )$ $\downarrow$	AUC@10 $\uparrow$	AVG $\epsilon (\lambda )$ $\downarrow$	MED $\epsilon (\lambda )$ $\downarrow$	AVG $\xi (f)$ $\downarrow$	MED $\xi (f)$ $\downarrow$	Time (ms) $\downarrow$
$\lambda _1 = \lambda _2$	9pt $\textbf{F} \lambda _1\lambda _2$	$\textbf{R},\vec {t},f,\lambda$	✗	45.33	10.89	0.30	0.92	0.82	0.33	0.15	152
	10pt $\textbf{F} \lambda _1\lambda _2$	$\textbf{R},\vec {t},f,\lambda$	✗	37.14	7.92	0.35	1.01	0.84	0.25	0.11	56
	8pt $\textbf{F} \lambda$	$\textbf{R},\vec {t},f,\lambda$	✗	26.07	5.15	0.47	0.98	0.84	0.30	0.07	106
	9pt $\textbf{F} \lambda$	$\textbf{R},\vec {t},f,\lambda$	✗	25.64	5.28	0.46	0.97	0.84	0.30	0.08	63
	7pt $\textbf{F}$	$\textbf{R},\vec {t},f$	$\lambda$ = 0	46.69	28.37	0.05	**0.00**	**0.00**	1.29	0.98	***53***
	7pt $\textbf{F}$	$\textbf{R},\vec {t},f,\lambda$	$\lambda = 0$	37.53	9.08	0.33	0.92	0.82	0.31	0.14	**50**
	7pt $\textbf{F}$	$\textbf{R},\vec {t},f,\lambda$	$\lambda \in \{0.0, -0.6, -1.2\}$	25.98	6.63	0.39	0.95	0.84	***0.24***	0.10	72
	6pt $\textbf{E}f$	$\textbf{R},\vec {t},f,\lambda$	$\lambda = 0$	21.82	5.21	0.47	0.85	0.83	0.38	0.08	58
	6pt $\textbf{E}f$	$\textbf{R},\vec {t},f,\lambda$	$\lambda \in \{0.0, -0.6, -1.2\}$	**15.70**	**4.28**	**0.53**	0.93	0.85	0.26	**0.06**	76
	7pt $\textbf{F}$	$\textbf{R},\vec {t},f$	GeoCalib - $\lambda$	30.34	10.48	0.28	0.66	0.67	0.39	0.19	(48)418
	7pt $\textbf{F}$	$\textbf{R},\vec {t},f,\lambda$	GeoCalib - $\lambda$	29.09	6.95	0.38	0.94	0.83	0.25	0.11	(51)421
	6pt $\textbf{E}f$	$\textbf{R},\vec {t},f$	GeoCalib - $\lambda$	18.34	5.46	0.46	0.76	0.79	0.36	0.09	(56)426
	6pt $\textbf{E}f$	$\textbf{R},\vec {t},f,\lambda$	GeoCalib - $\lambda$	***16.62***	***4.43***	***0.52***	0.91	0.84	0.27	***0.06***	(58)428
	5pt $\textbf{E}$	$\textbf{R}, \vec {t}$	GeoCalib - $\lambda ,f$	17.41	5.97	0.42	***0.57***	***0.60***	0.26	0.16	(24)394
	5pt $\textbf{E}$	$\textbf{R},\vec {t},f,\lambda$	GeoCalib - $\lambda ,f$	20.17	6.12	0.42	0.88	0.83	**0.23**	0.10	(66)436
	3pt $\textbf{E}$	$\textbf{R},\vec {t}$	GeoCalib - $\lambda ,f,\vec {g}$	23.43	6.29	0.40	0.57	0.60	0.26	0.16	(22)392
	3pt $\textbf{E}$	$\textbf{R},\vec {t},f,\lambda$	GeoCalib - $\lambda ,f,\vec {g}$	30.59	6.78	0.38	0.88	0.83	0.25	0.11	(65)435

**Fig. 6:**
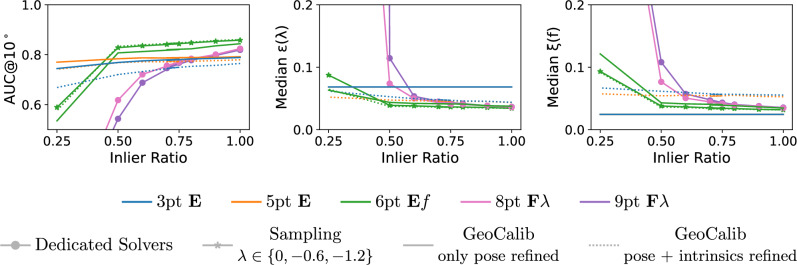
Pose AUC@10$^{\circ }$, mean absolute $\lambda$ errors, and relative focal length errors plotted for different synthetically induced inlier ratios for the case of shared intrinsics ($\lambda _1 = \lambda _2$) on the CATHEDRAL dataset

**Fig. 7:**
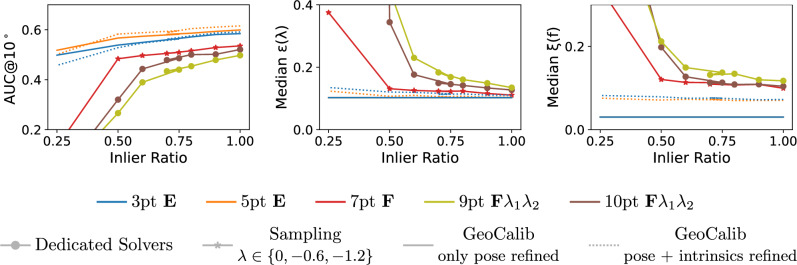
Pose AUC@10$^\circ$, mean absolute $\lambda$ errors, and relative focal length errors plotted for different synthetically induced inlier ratios for the case of different intrinsics ($\lambda _1 \ne \lambda _2$) on the CATHEDRAL dataset

#### Scenario C - *Visible distortion*

In the last scenario, we assume that we know that our images have visible (but unknown) distortion. To simulate this, we distort the images with distortions corresponding to undistortion parameters uniformly sampled from the interval $[-1.8, -0.5]$. For the sampling-based strategy, we tested two different variants: (1) $\textbf{U}_1 = \textbf{U}_2 = \{-0.9\}$ and (2) $\textbf{U}_1 = \textbf{U}_2 = \{-0.6,-0.9,-1.2\}$. The results are shown in Tab. 3.

For the case of shared intrinsics ($\lambda _1 = \lambda _2$) the 6pt $\textbf{E}f$ solver with the sampling strategy performs the best. For the case of two different cameras ($\lambda _1 \ne \lambda _2$) the strategy using a combination of GeoCalib predictions, the 5pt $\textbf{E}$ solver and refinement of intrinsics in LO performs the best. Again, for both cases the proposed sampling-based and learning-based prior strategies perform significantly better than the dedicated radial distortion solvers.

**Fig. 8:**
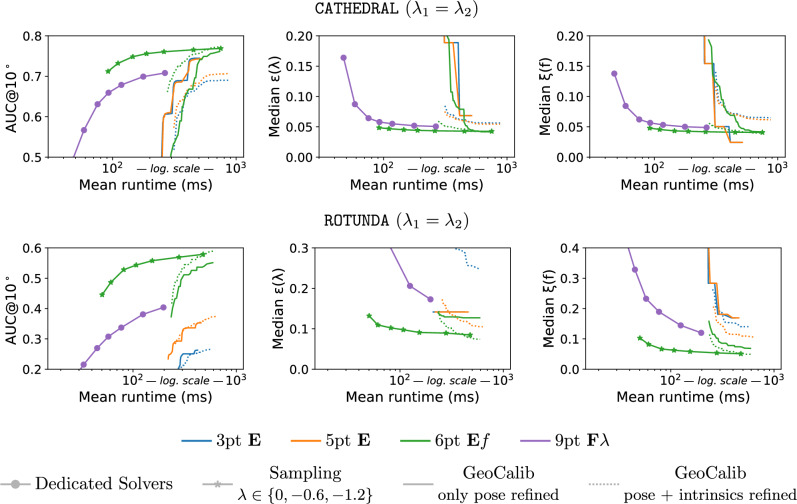
Pose AUC@10$^\circ$, mean absolute $\lambda$ errors and relative focal length errors plotted for different total runtimes of the compared methods. We consider the situation when the cameras have equal intrinsics ($\lambda _1 = \lambda _2$). For all methods we vary the total number of RANSAC iterations ($\{10, 20, 50, 100, 200, 500, 1000\}$). For the methods utilizing the learning-based prior strategy with GeoCalib we also vary the total number of LM iterations to produce the final estimate ($\{1, 2, 5, 30\}$). To plot the curves we always take the best performing setting achieving equal or shorter runtime. The dedicated 8pt $\textbf{F} \lambda$ minimal solver is not shown, since given the same time budget it always performs worse than the non-minimal 9pt $\textbf{F} \lambda$ solver.

**Fig. 9:**
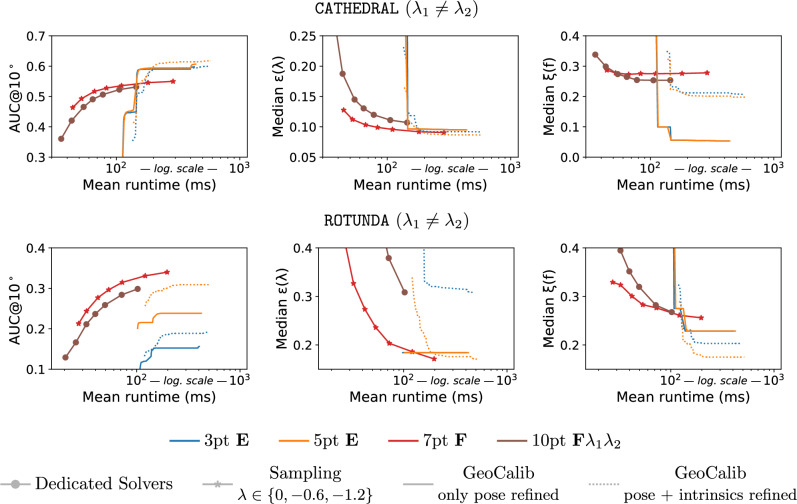
Pose AUC@10$^\circ$, mean absolute $\lambda$ errors and relative focal length errors plotted for different total runtimes of the compared methods. We consider the situation of two different cameras ($\lambda _1 \ne \lambda _2$). For all methods we vary the total number of RANSAC iterations ($\{10, 20, 50, 100, 200, 500, 1000\}$). For the methods utilizing the learning-based prior strategy with GeoCalib we also vary the total number of LM iterations to produce the final estimate ($\{1, 2, 5, 30\}$). To plot the curves we always take the best performing setting achieving equal or shorter runtime. The dedicated 9pt $\textbf{F} \lambda _1\lambda _2$ minimal solver is not shown, since given the same time budget it always performs worse than the non-minimal 10pt $\textbf{F} \lambda _1\lambda _2$ solver

#### Natural Scenes

We repeat the three scenarios, but instead of the ETH3D dataset we use the PragueParks dataset. The results are shown in Tabs. 4, 5 and 6. This dataset contains natural scenes. For such scenes the GeoCalib network produces worse intrinsics estimates leading to a worse overall performance of the learning-based prior strategy, especially when visible distortion is present, *i.e.*in scenarios A and C. We note that for the case of shared intrinsics the 6pt $\textbf{E}f$ solver with the sampling strategy leads to the best results. While for the case of two different cameras the dedicated non-minimal 10pt $\textbf{F} \lambda _1\lambda _2$ solver performs the best with the sampling strategy combined with the 7pt $\textbf{F}$ solver achieving slightly worse results.

These experiments on both ETH3D and PragueParks datasets show some important observations: (1) The sampling-based and learning-based prior strategies perform similar to or even better than the dedicated minimal radial distortion solvers. (2) Having additional knowledge about the cameras (even vague knowledge, *e.g.*, that the cameras have visible distortion) can improve the performance of the sampling-based strategy. (3) The sampling-based strategy with the 6pt $\textbf{E}f$ solver outperforms the learning-based prior strategy when the cameras can be assumed to have the same intrinsics ($\lambda _1 = \lambda _2$). For the case of two different cameras ($\lambda _1 \ne \lambda _2$), using the GeoCalib predictions may lead to significantly better results than relying on a simple sampling strategy when considering images of man-made structures. (4) In general, the sampling- based approach is more robust than the one using learning-based priors and works well for all tested datasets.

### Effect of outliers

The inlier ratio among the point correspondences impacts the performance of the solvers. This effect is particularly pronounced for solvers that require a larger number of point correspondences. To evaluate the impact of the inlier ratio on the accuracy of the tested approaches, we conduct a semi-synthetic experiment using the CATHEDRAL scene from our own dataset. To control the inlier ratio, we first filter out all point correspondences which would be considered outliers with respect to the ground truth intrinsics and poses, resulting in a set of real inlier-only correspondences. Then, we introduce spurious synthetic point correspondences by adding uniformly distributed points in both images. The number of added spurious correspondences determines the final inlier ratio. We evaluate the selected methods across varying inlier ratios using RANSAC with early termination for a maximum of 10,000 iterations. The results for the case of shared intrinsics ($\lambda _1 = \lambda _2$) and different intrinsics ($\lambda _1 \ne \lambda _2$) are shown in Fig. 6 and Fig. 7, respectively. The results show that the dedicated solvers perform significantly worse at lower inlier ratios. This is expected as the solvers have a significantly lower chance of finding all-inlier samples compared to the alternatives since they require more point correspondences to estimate their models. We note that this comparison introduces outliers to the point correspondences while the predictions from GeoCalib remain unaffected. Still, the experiment reveals a failure case for dedicated solvers, where sampling- and learning-based strategies achieve superior performance.

### Real-World Scenario

In the previous experiments, we synthesized distortions to be able to precisely measure the behavior of the different approaches under varying levels of distortion. To evaluate the performance of the tested methods under real-world conditions and for cameras with different distortions, we use the EuRoC-MAV dataset and our own dataset consisting of the ROTUNDA and CATHEDRAL scenes. Results are shown in Tab. 7 for ROTUNDA, Tab. 8 for CATHEDRAL and Tab. 9 for EuRoC-MAV. These results show that the sampling-based and learning-based prior strategies significantly outperform the dedicated solvers. For the equal camera case ($\lambda _1 = \lambda _2$) the 6pt $\textbf{E}f$ solver with LO of intrinsics achieves best results when used with both sampling $\textbf{U}_1 = \textbf{U}_2 = \{0,-0.6,-1.2\}$ and GeoCalib for all scenes. We note that for both of these approaches the resulting accuracy is very similar. The sampling strategy results in increased RANSAC time, but this increase is lower than the time required for GeoCalib inference (see Sec. 4.7.1).

For the case of two different cameras ($\lambda _1 \ne \lambda _2$), the learning-based prior strategy performs the best for the CATHEDRAL scene while the sampling-based strategy with the 7pt $\textbf{F}$ solver and $\textbf{U}_1 = \textbf{U}_2 = \{0,-0.6,-1.2\}$ provides the best pose estimates for the ROTUNDA scene. We note that all methods perform worse on the ROTUNDA scene in terms of both the estimated poses and intrinsics suggesting that this scene is significantly more difficult than CATHEDRAL. This can also be a reason why GeoCalib’s intrinsics estimates are less accurate for ROTUNDA than for CATHEDRAL, resulting in worse performance of the learning-based prior strategy compared to the sampling-based strategy in the ROTUNDA different distortion scenario.

We also note that the GeoCalib predictions of focal lengths for the CATHEDRAL scene are significantly better than relying on the geometry obtained from point correspondences via solvers. Similarly, when GeoCalib is used with refinement of the intrinsics in LO, the focal length error increases significantly, while the pose estimate becomes more accurate. This may be caused by degenerate camera configurations which introduce ambiguity into focal length estimation (Bougnoux, 1998). In such configurations, the single-view predictions of intrinsics based on learned visual features and geometric cues may be more accurate than relying on point correspondences and epipolar geometry.

#### Speed-accuracy trade-off

In some situations, the time for relative pose estimation is limited. To evaluate the viability of the different methods in such scenario we conduct an experiment on both scenes to assess each solver’s performance, in terms of the AUC@10$^\circ$ of pose errors, and the median absolute error of the estimated undistortion parameter(s) and focal length(s), for different numbers of RANSAC iterations. For the learning-based prior strategy we also varied the total number of LM iterations to obtain the final intrinsics estimate. We evaluate the runtimes using a one core of the 2 GHz Intel Xeon Gold 6338 CPU for RANSAC and an A100 GPU for GeoCalib inference. Fig. 8 shows the plots of the measured metrics vs. the average run-time for the case of shared intrinsics ($\lambda _1 = \lambda _2$). The results show that the 6pt $\textbf{E}f$ solver combined with the sampling strategy provides the best speed-accuracy tradeoff in most of the tested budgets. Fig. 9 shows the same plots for the case of two different cameras ($\lambda _1 \ne \lambda _2$). In this case the 7pt solver with sampling performs best in situations low time budget (<100 ms). When more time is available for computation the learning-based prior strategy may be more optimal.

We note that the hardware setup used for our experiments pairs a powerful GPU (A100) with a CPU core that is slower than mid-tier CPUs available on the market. In practice, especially when used on edge devices, the runtime for GeoCalib may be significantly larger than 120+ ms making its speed-accuracy tradeoff worse compared to the CPU-only approaches in such scenarios.

## Conclusion

Modeling radial distortion during relative pose estimation is important. Yet, (minimal) radial distortion solvers are significantly more complex than solvers for pinhole cameras, in terms of both runtime and implementation efforts. This paper thus asks the question whether minimal radial distortion solvers are actually necessary in practice. To answer this question, we considered two approaches that do not require minimal radial distortion solvers: The first samples radial distortion parameters from a fixed set of values rather than estimating them, while the second uses a neural network to predict the parameters. Both approaches uses the sampled / predicted parameters in combination with a pinhole relative pose solver. Extensive experiments show that both of these simple strategies outperforms existing distortion solvers. Both approaches are easy to implement and faster than the best-performing minimal distortion solvers. Moreover, on real data, they result in better accuracy than the dedicated radial distortion solvers. We conclude that the dedicated distortion solvers are not truly necessary in practice. We also show that the sampling-based approach is more robust than the one using learning-based priors and works well for all tested datasets, despite not requiring a GPU.

## Data Availability

The code for the methods and evaluation, along with the new benchmark dataset are available at https://github.com/kocurvik/rdnet.

## Footnotes

1 A point in one image maps to an epipolar *curve* in a radially distorted second image. Ignoring radial distortion thus means approximating this curve by a line. The approximation is only good around the center of strongly distorted images.

2 Usually one more point for cameras with a common unknown radial distortion and two more points for cameras with different radial distortions.

3 Instead of $\lambda$ and $\lambda ^{\prime }$ as used in (1), we use $\lambda _1$ and $\lambda _2$ for better readability.
